# Mechanism of Diiron Hydrogenase Complexes Controlled by Nature of Bridging Dithiolate Ligand

**DOI:** 10.1002/open.202100238

**Published:** 2022-01-04

**Authors:** Mookan Natarajan, Naveen Kumar, Meenakshi Joshi, Matthias Stein, Sandeep Kaur‐Ghumaan

**Affiliations:** ^1^ Department of Chemistry University of Delhi Delhi 110007 India; ^2^ Max-Planck-Institute for Dynamics of Complex Technical Systems Molecular Simulations and Design Group Sandtorstrasse 1 39106 Magdeburg Germany

**Keywords:** bio-inorganic chemistry, DFT, electrocatalysis, hydrogenases, hydrogen evolution

## Abstract

Bio‐inorganic complexes inspired by hydrogenase enzymes are designed to catalyze the hydrogen evolution reaction (HER). A series of new diiron hydrogenase mimic complexes with one or two terminal tris(4‐methoxyphenyl)phosphine and different μ‐bridging dithiolate ligands and show catalytic activity towards electrochemical proton reduction in the presence of weak and strong acids. A series of propane‐ and benzene‐dithiolato‐bridged complexes was synthesized, crystallized, and characterized by various spectroscopic techniques and quantum chemical calculations. Their electrochemical properties as well as the detailed reaction mechanisms of the HER are elucidated by density functional theory (DFT) methods. The nature of the μ‐bridging dithiolate is critically controlling the reaction and performance of the HER of the complexes. In contrast, terminal phosphine ligands have no significant effects on redox activities and mechanism. Mono‐ or di‐substituted propane‐dithiolate complexes afford a sequential reduction (electrochemical; E) and protonation (chemical; C) mechanism (ECEC), while the μ‐benzene dithiolate complexes follow a different reaction mechanism and are more efficient HER catalysts.

## Graphical abstract

**A series of homo‐nuclear iron complexes** shows good catalytic performance in the hydrogen evolution reaction. Systematic variations of terminal and bridging ligands allow dissecting their individual contributions to the reaction mechanism. In particular, the nature of the μ‐bridging thiolate is found to be critical.

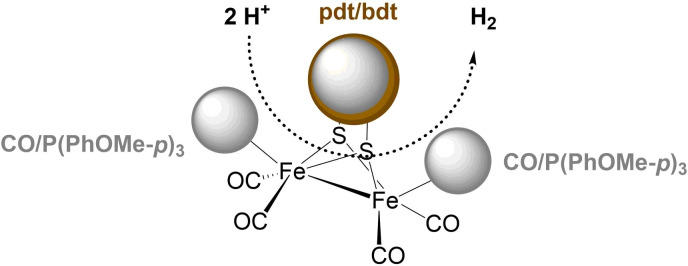

## Introduction

The [FeFe] hydrogenase (H_2_ase) is Nature's most efficient and inexpensive catalyst for hydrogen production.[^1^, ^2^, ^3^, ^4^, ^5^, ^6^] From protein crystal structure analysis, it is known that the active site of this H_2_ase features a butterfly [2Fe2S] core (the H‐cluster).[^7^, ^8^] The two iron atoms in the H‐cluster are coordinated by small inorganic carbonyl (CO)/cyanide (CN) ligands, a μ‐bridging aza‐dithiolate and a cysteinyl‐S‐linked cubic 4Fe4S cluster. Bio‐inorganic and bio‐mimetic iron‐sulfur complexes take up structural or functional principles from the enzyme's active site which has led to the synthesis of a large number of model complexes as mimics of the enzyme. Particular focus was laid on the nature of dithiolate linkers with different bridgehead groups −CH_2_‐X−CH_2_− with X=CH_2_, NH, S, O[^9^, ^10^, ^11^, ^12^, ^13^, ^14^, ^15^, ^16^] or on modified linkers such as dichalcodenolates.[^17^, ^18^, ^19^, ^20^, ^21^, ^22^, ^23^]

The structural aspects have also been studied by the substitution of terminal CO ligands with electron‐donating ligands, like cyanides and phosphines.[^24^, ^25^, ^26^, ^27^, ^28^, ^29^, ^30^, ^31^] In model complexes, terminal phosphine ligands are good substitutes for naturally occurring CN^−^.[^32^, ^33^] The bridging di‐thiolates play an important role in the development of the biomimetic chemistry of [FeFe] hydrogenase.[^34^, ^35^, ^36^, ^37^] Here, we extend previous work on propane dithiolate (pdt)‐bridged di‐nuclear iron‐sulfur complexes[^38^, ^39^, ^40^] with terminal mono‐ or di‐substituted tris(4‐methoxyphenyl)phosphine (P(PhOMe‐*p*)_3_) ligands. The introduction of an aromatic benzene di‐thiolate (bdt) allows the detailed investigation of the effects of the terminal phosphines and the nature of the μ‐bridging ligands on complex stability and catalytic performance.

We report the synthesis, spectroscopic characterization and electrochemical investigation plus DFT calculations for complexes [Fe_2_(μ‐pdt)(CO)_5_(P(PhOMe‐*p*)_3_)] **1**, [Fe_2_(μ‐pdt)(CO)_4_(P(PhOMe‐*p*)_3_)_2_] **2**, [Fe_2_(μ‐bdt)(CO)_5_(P(PhOMe‐*p*)_3_)] **3** and [Fe_2_(μ‐bdt)(CO)_4_(P(PhOMe‐*p*)_3_)_2_] **4** in the presence of acetic, trifluoroacetic and perchloric acids. Further, we have investigated the change and control of catalytic behavior of these diiron complexes upon modifying the bridgehead group from aliphatic to aromatic and terminal ligands from CO to phosphine.

## Results and Discussion

### Synthesis and Characterization of Di‐nuclear {FeFe} Complexes 1–4

**Scheme 1:**
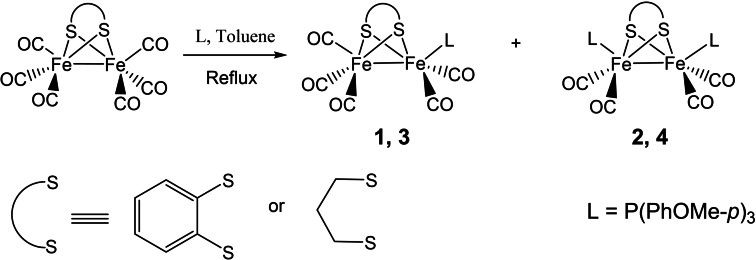
Scheme for the synthesis of complexes **2**–**4**.

The starting materials [Fe_2_(μ‐pdt)(CO)_6_] **A** and [Fe_2_(μ‐bdt)(CO)_6_] **B** (pdt=propanedithiolate and bdt=1,2‐benzenedithiolate) and complex **1** were synthesized as reported in literature.[^41^, ^42^, ^43^] Complexes **2**–**4** were prepared from the hexacarbonyl precursors **A** and **B** and the phosphine ligand (L=P(PhOMe‐*p*)_3_) by refluxing in toluene under argon atmosphere for 4–5 h (Scheme 1). The complexes were purified by column chromatography and recrystallized from *n*‐hexane‐dichloromethane solutions.

**Figure 1:**
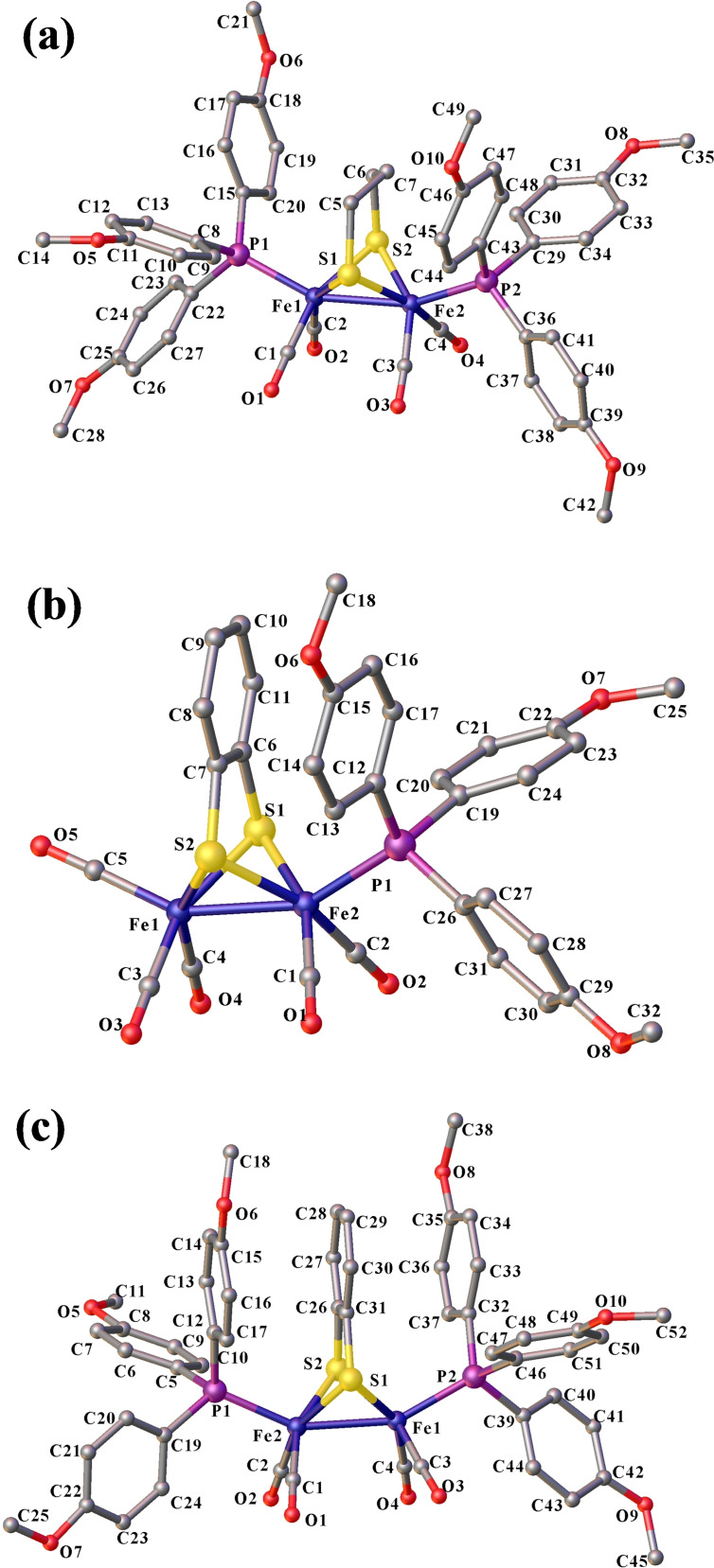
Molecular structures of (a) [Fe_2_(μ‐pdt)(CO)_4_(P(PhOMe‐*p*)_3_)_2_] **2**, (b) [Fe_2_(μ‐bdt)(CO)_5_(P(PhOMe‐*p*)_3_)] **3** and (c) [Fe_2_(μ‐bdt)(CO)_4_(P(PhOMe‐*p*)_3_)_2_] **4** in the solid state. Hydrogen atoms have been omitted for clarity.

The molecular structures of **2**, **3** and **4** are shown in Figure 1. The crystallographic parameters for complexes **2**–**4** are summarized in Table S1 together with selected bond lengths and bond angles of complexes **2**–**4** in Tables S2–S3 in the Supporting Information.

Furthermore, all four complexes **1**–**4** were structurally optimized using Density Functional Theory (DFT) approaches both in the absence and presence of a solvent (acetonitrile, see Experimental Section). The optimized structural parameters of complexes **2**–**4** are reported in Tables S4 and S5 (see Supporting Information). The calculated results, using the BP86 GGA functional, are in good agreement with the experimental results and are reported in the main article. B3LYP results are given in the Supporting Information.

In complexes **1**–**4**, the bond angles reveal a distorted square pyramidal coordination geometry around both iron centers and the [2Fe2S] core of the complexes has the expected butterfly conformation, as reported previously for analogous diiron(I) pdt and bdt complexes.[^43^, ^44^, ^45^, ^46^, ^47^, ^48^, ^49^, ^50^, ^51^, ^52^, ^53^, ^54^, ^55^, ^56^, ^57^] The phosphine ligands occupy the apical position in the investigated diiron complexes which is consistent with the configurations seen for other reported PR_3_‐monosubstituted complexes.[^3^, ^44^, ^45^, ^52^, ^54^, ^55^, ^58^, ^59^] The Fe−Fe bond distances in **2, 3** and **4** are 2.53, 2.50 and 2.50 Å, respectively, thus only slightly shorter than the distance found in the naturally occurring [FeFe] hydrogenase enzymes (2.55‐2.60 Å).[^7^, ^8^] A Fe−Fe bond distance of 2.52 Å was reported for complex **1**.^[43]^ The calculated Fe−Fe bond distances in complexes **1**, **2**, **3** and **4** are 2.55, 2.54, 2.51 and 2.50 Å, respectively, which is in excellent agreement with the corresponding experimental values. Stronger σ‐donor properties of the phosphine ligand as compared to the carbonyl group are responsible for the slightly different Fe−Fe bond lengths. The Fe−Fe bond length in the studied complexes **2**–**4** is similar to that in the related compounds containing apical phosphine ligands.[^3^, ^58^, ^59^] The phosphine ligand coordinated to one of the iron atoms in the complexes **2**–**4** induces a slight asymmetry of the [2Fe2S] skeleton which is reflected by shorter Fe−S bonds to the moiety. As also reported for **1**, the average Fe‐C_CO_ distances of the phosphine coordinated iron center in complexes **2**–**4** are slightly shorter for the all carbonyl‐coordinated iron atoms (Tables S2‐S5, see Supporting Information). Introduction of the sterically demanding terminal phosphine only has a minor effect on the Fe‐Fe−L (L=CO, PR_3_) bond angle (in **1** the Fe(2)‐Fe(1)‐P(1) angle is 8.47° larger than the Fe(1)‐Fe(2)‐C(5) angle).^[43]^ The change in bond angle is even smaller (1.5°) in mono‐ and di‐substituted bdt complexes **3** and **4** (P(1)‐Fe(2)‐Fe(1), 153.0° in **4** and C(5)‐Fe(1)‐Fe(2), 151.5° in **3**). The DFT‐optimized structural parameters agree well with those found in crystal structures of complexes **2**, **3** and **4** and are reported in Tables S2‐S5.

The three complexes **2**–**4** were further characterized by FTIR and NMR spectroscopy and density functional theory calculations. The FTIR spectra of the complexes **2**–**4** are provided in Figures S1‐S2 (Supporting Information). The mono‐substituted complexes (**1** and **3**) display strong absorption bands between 2037–1926 cm^−1^ that are assigned to the terminal carbonyl groups (Table S6, see Supporting Information). For the di‐substituted complexes (**2** and **4**), the absorption bands are observed in the region 1988–1890 cm^−1^ (Table S6). The calculated IR spectra agree well with the experiment and show strong ν_CO_ absorption bands for the terminal carbonyl ligands between 2038–1945 cm^−1^ (for **1** and **3**) and 1997–1926 cm^−1^ (for **2** and **4**) (Figures S3–S4, see Supporting Information). The ν_CO_ bands in these complexes are shifted towards lower wavenumbers in comparison to the hexacarbonyl analogues[^41^, ^42^] but are similar to those observed for the analogous phosphine‐substituted complexes.[^3^, ^41^, ^58^] The shift could be due to the attachment of a more basic phosphine ligand on one of the iron centers. The calculated shift in ν_CO_ bands with respect to the all‐carbonyl complex is 20–40 cm^−1^ for the mono‐ (**1** and **3**) and 55–70 cm^−1^ for the di‐substituted (**2** and **4**) complexes and is in agreement with the phosphine ligands being stronger σ‐donors. Description of the NMR data for complexes **2**–**4** is given in the Supporting Information (Figures S5–S8).

### Electrochemical Studies

#### Cyclic Voltammetry in Absence of Proton Source

Cyclic voltammograms (CVs) for complexes **1**–**4** were measured in acetonitrile under an argon atmosphere (see Table 1). The CVs for **1** display two irreversible reduction events at *E*_pc_=−1.87 and −2.37 V and two irreversible oxidations at *E*_pa_=0.35 and 0.70 V (Figure S9, see Supporting Information). On the other hand, only one irreversible reduction at −1.65 V is observed for complex **3** (Figure S9). The reductions for complexes **1** and **3** occur at more negative potentials than for the all‐carbonyl complex due to the substitution of one CO ligand with the electron‐donating phosphine ligand.[^42^, ^44^, ^60^] Also, the reduction of **3** occurs at a less negative potential and its oxidation at a more positive potential than **1** due to the aromatic bdt ligand in **3**. The electrochemical data are consistent with the results of similar model complexes reported in the literature.^[58b]^

**Table 1:** Experimental and calculated electrochemical data for complexes **1**–**4**.

Complex	*E*_pc_/V	*E*_pa_/V	*E*_cat_/V^[c]^	Calculated *E*_pc_/V^[d]^
**1**	−1.87	0.35	−1.79	−1.81
	−2.37	0.70	−1.96	−2.46
**2**	−2.10	0.27	−1.65	−2.37
	−2.51	–	−1.99	−2.60
**3**	−1.65	0.49	−1.57	−1.60
	–	–	−1.91	−1.77
**4**	−2.02	0.39	−1.43	−1.90
	–	0.67	−1.71	−2.09
[Fe_2_(CO)_6_(μ‐pdt)]	−1.74^[a]^	–	–	−1.48
	−2.35^[a]^	–	–	−2.15
[Fe_2_(CO)_6_(μ‐bdt)]	−1.31^[b]^	–	–	−1.32

[a] In dichloromethane; [b]$E_{1/2}^{\mathrm{red}}$ ; [c] in TFA; [d] BP86D3/def2‐TZVP(COSMO).

The CVs for the di‐substituted complex **2** display two irreversible reduction waves at *E*_pc_=−2.10 and −2.51 V while only one irreversible reduction peak was observed at −2.02 V for complex **4** (Figure S10, see Supporting Information). The potentials have shifted to more negative values in comparison to **1** and **3** due to the presence of two phosphine ligands. The cyclic voltammograms for complexes **1**–**4** were also measured at different scan rates (50‐1000 mV s^−1^); the peak current of the reduction waves was proportional to the square root of the scan rate thus, indicating that the electrochemical processes were diffusion‐controlled (Figure S11, see Supporting Information).^[61]^

The calculated reduction potentials of the complexes **1**–**4** relative to Fc^0/+^ match very well with the corresponding experimental values as reported in Table 1. B3LYP results are given in the Supporting Information and are deviating more from experimental values (Table S7). The reduction potential becomes more negative with the replacement of CO ligands with σ‐donor phosphine ligands. Therefore, for the di‐substituted complexes (**2** and **4**), the first one‐electron reduction occurs at more negative potentials as compared to that for the mono‐substituted counterparts **1** and **3** (see Table 1).

In the mono‐reduced pdt‐bridged complexes **1^−^** and **2^−^**, the unpaired spin density is delocalized over the Fe metal atoms, while in the reduced bdt‐bridged complexes **3^−^** and **4^−^**, the spin density is localized on one Fe atom only.

It is noteworthy that, upon reduction of the pdt‐bridged complexes **1** and **2**, all Fe−S bonds of the reduced species **1^−^** and **2^−^**, increase in length (2.28/2.35 Å in **1**/**1^−^** and 2.29/2.33 Å in **2**/**2^−^**) but remain intact. The Fe−Fe bond length slightly changes from 2.55 Å in **1** to 2.87 Å in **1^−^** and from 2.54 Å in **2** to 2.67 Å in **2^−^**.

In the reduced bdt‐bridged species **3^−^** and **4^−^**, however, one of the Fe−S bonds breaks and Fe⋅⋅⋅S distances increase significantly (from 2.31 Å to 3.06 Å (in **3**→**3^−^**); from 2.32 Å to 3.60 Å (in **4**→**4^−^**)). This opening provides an accessible site for protonation (see below). However, no significant changes are observed in the Fe−Fe bond distances (2.51 Å to 2.56 Å in **3**/**3^−^** and 2.50 Å to 2.58 Å in **4**/**4^−^**).

It is worth mentioning that the unpaired electron spin density in **3^−^** and **4^−^** is only localized at the Fe atom with the open coordination site. Since the aromatic bdt‐bridged reduced species (**3^−^** and **4^−^**) possess an accessible coordination site, proton reduction would be easier in complexes **3** and **4** as compared to that in complexes **1** and **2**. The Gibbs free energy change (ΔG) for the first one‐electron reduction (E) of **1/1^−^** (−298 kJ mol^−1^) and **2/2^−^** (−244 kJ mol^−1^) is less negative than that of **3/3^−^** (−318 kJ mol^−1^) and **4/4^−^** (−290 kJ mol^−1^). We can thus discriminate the effect of mono‐ versus di‐substitution (+54 kJ mol^−1^ for pdt and +28 kJ mol^−1^ for bdt complexes) and substitution of an alkyl for an aromatic di‐thiolate ligand (−20 kJ mol^−1^ for mono‐ and −46 kJ mol^−1^ upon di‐substitution).

Also, the calculated first one‐electron reduction potentials of the bdt‐bridged complexes **3** (−1.60 V) and **4** (−1.90 V) are less negative than for the pdt‐bridged complexes **1** (−1.81 V) and **2** (−2.37 V). It is interesting to note that the second one‐electron reductions of **3^−^/3^2−^** (−1.77  V) and **4^−^/4^2−^** (−2.09 V) also occur at a low potential, while for the pdt‐bridged complexes **1^−^/1^2−^** (−2.46 V) and **2^−^/2^2−^** (−2.60 V), they appear to occur at higher potentials.

The close first and second one‐electron redox potentials in **3** and **4** may actually make a clear experimental assignment difficult. Therefore, for the hydrogen evolution reaction (HER), we investigate the EECC and ECEC reaction mechanisms for complexes **3** and **4**, while for complexes **1** and **2** only the ECEC reaction mechanism is proposed (where E=Electrochemical and C=Chemical).

#### Cyclic Voltammetry in Presence of Proton Source

Complexes **1**–**4** were examined with regard to their performance as electrocatalysts for the reduction of protons to molecular hydrogen in the presence of three different acids (acetic, trifluoroacetic (TFA) and perchloric acid (HClO_4_)). The pdt‐bridged complexes **1** and **2** show no catalytic activity in the presence of the weak acetic acid due to the high p*K*_a_ value of the acid in comparison to the one‐electron reduced species.^[62]^

**Figure 2:**
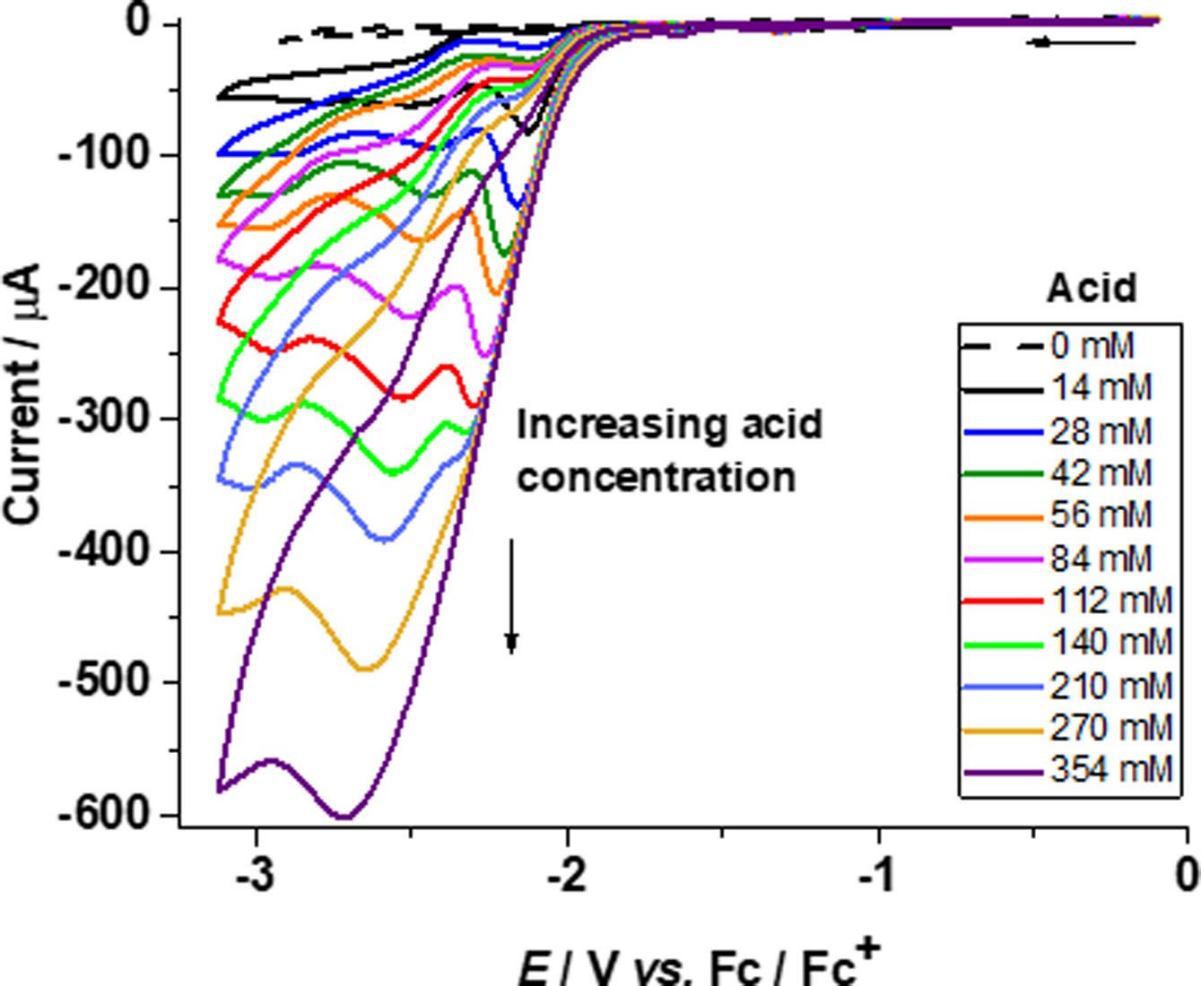
Cyclic voltammograms for complex **3** (1.11 mm) in acetonitrile without acid (– ‐) and with increasing amounts (14–354 mm) of acetic acid (—) at a scan rate of 0.1 V s^−1^.

CVs of **3** in the presence of acetic acid display a new peak at −2.12 V versus Fc/Fc^+^, which shifts cathodically with the increase in the amount of acid (Figure 2). In addition, a second peak is observed at −2.43 V. The new peaks appear at a value more negative than the reduction of the complex **3** at −1.65 V in the absence of acid. The peak at −2.12 V disappears completely after adding 354 mm of acid, while the second peak at −2.43 V persists at higher concentrations of acid in solution.

**Figure 3:**
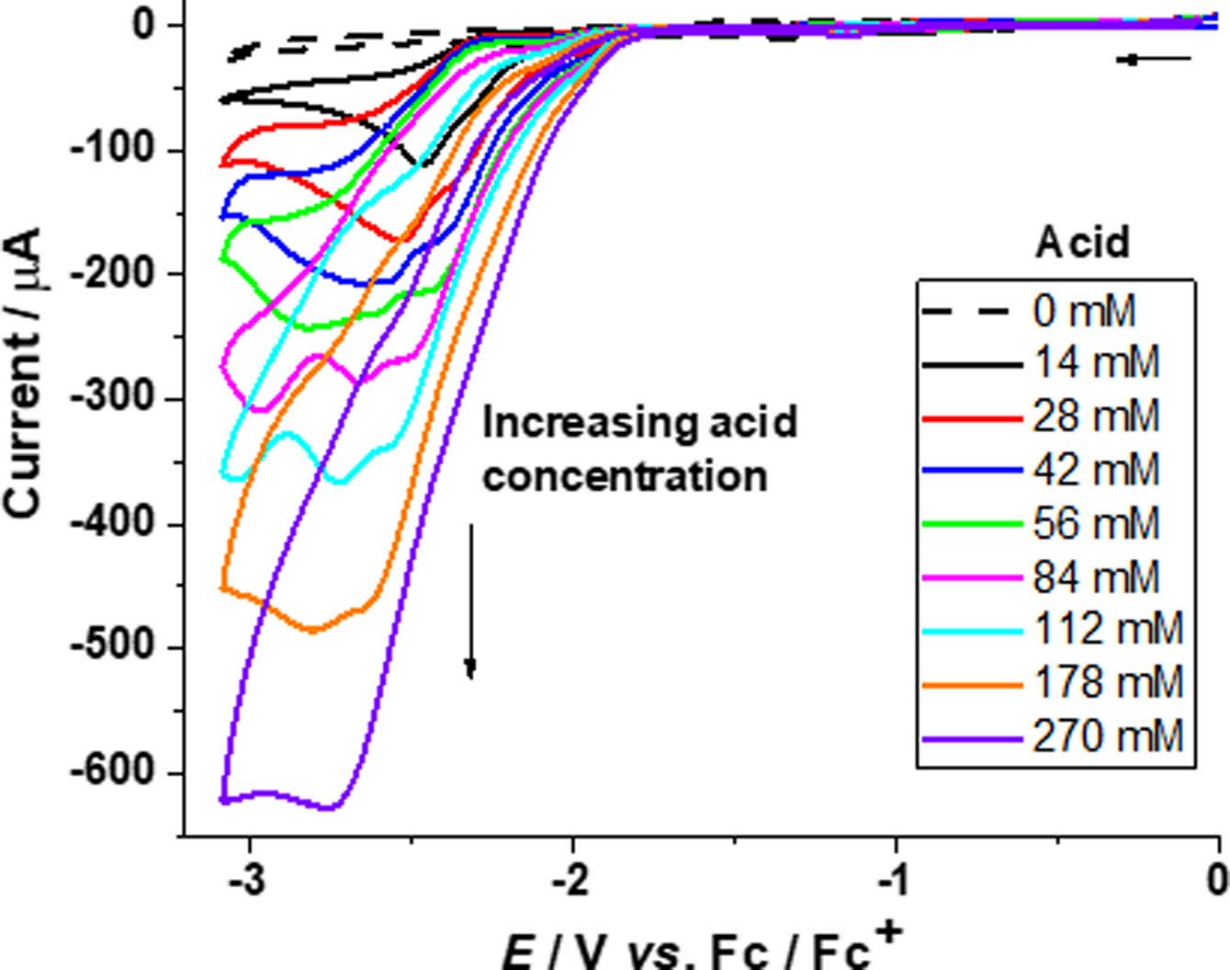
Cyclic voltammograms for complex **4** (1.13 mm) in acetonitrile without acid (– ‐) and with increasing amounts (14–270 mm) of acetic acid (—) at a scan rate of 0.1 V s^−1^.

The increase in current paralleling the increasing amount of acid can be attributed to the reduction of protons yielding molecular hydrogen.^[58b]^ For complex **4**, a peak is first observed at −2.46 V with the appearance of a second peak at −2.36 V upon adding 28 mm of acid, which shifts cathodically with the increase of acid concentration in the solution (Figure 3). Subsequently, only one peak remains up to 270 mm of acid in the solution. This indicates that two catalytic reaction mechanisms may be occurring simultaneously, that is, an ECEC and an EECC mechanism.

**Figure 4:**
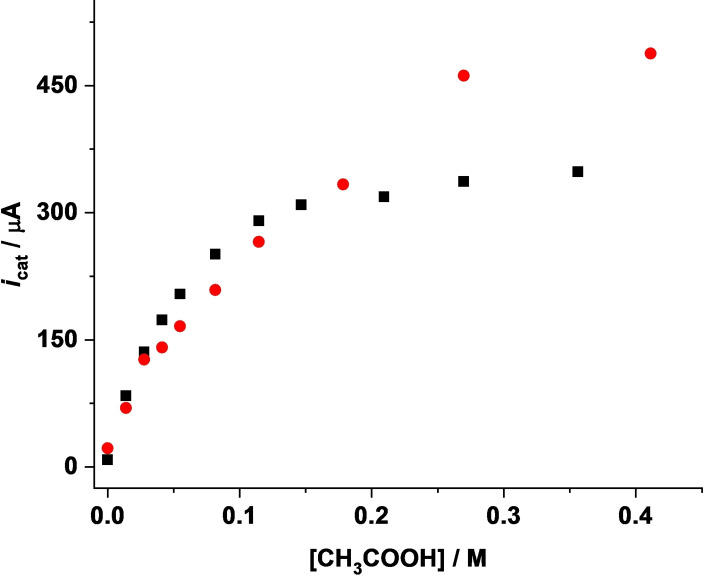
Plots of *i*_cat_/μA vs. [CH_3_COOH]/m for **3** (1.11 mm) (▪) and **4** (1.13 mm) (•) for the first reduction peak at a scan rate of 0.1 V s^−1^. The negative sign for the current has been ignored.

The CVs were corrected for the background currents without catalyst in the presence of acetic acid. (see Supporting Information, Figure S12). Hence, the acid‐induced currents in the presence of catalysts can be attributed to catalytic turnover.^[63]^ From the plot of peak currents (*i*_cat_) versus acid concentration for complexes **3** and **4**, it can be seen that the bdt‐bridged di‐substituted complex **4** is a slightly better catalyst than the mono‐substituted complex **3** in the presence of acetic acid (Figure 4 and Figure S13, Supporting Information). However, the overpotential for **3** (0.66 V) is lower than that of **4** (0.90 V).

**Figure 5:**
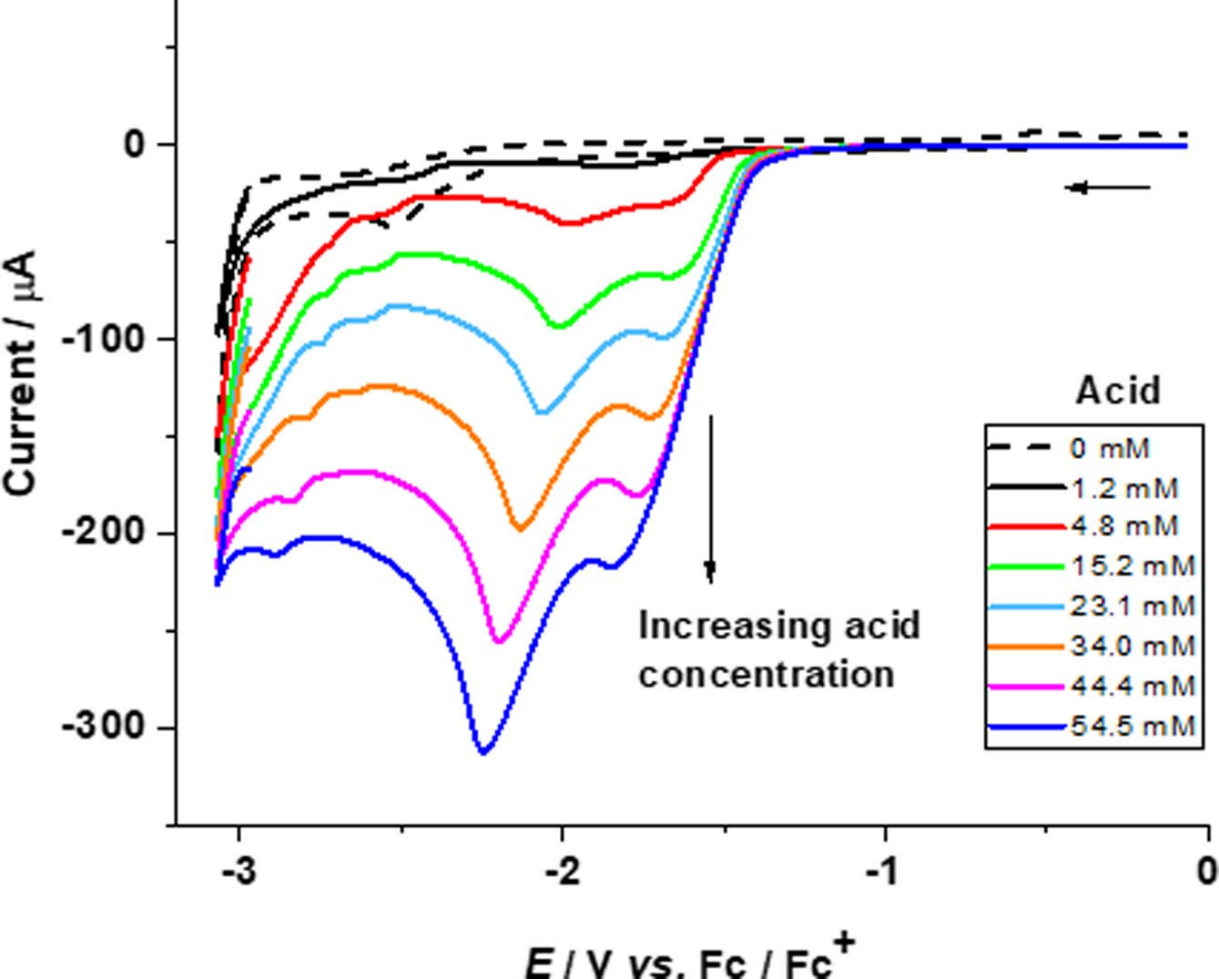
Cyclic voltammograms for complex **2** (0.67 mm) in acetonitrile without acid (– ‐) and with increasing amounts (1.2–54.5 mm) of TFA (—) at a scan rate of 0.1 V s^−1^. Reverse scans have been omitted for clarity.

Electrochemical investigations for the four complexes **1**–**4** were also performed in the presence of trifluoroacetic acid (TFA). A similar pattern in the CVs can be observed for all the four complexes in the presence of TFA (Figure 5 and Figures S14–S16, Supporting Information). The reduction potentials of the bdt‐bridged complexes in presence of TFA are lower than those of the aliphatic ones, hence leading to lower overpotentials. Moreover, the presence of two phosphine ligands further lowers the overpotential for the di‐substituted complexes.

**Figure 6:**
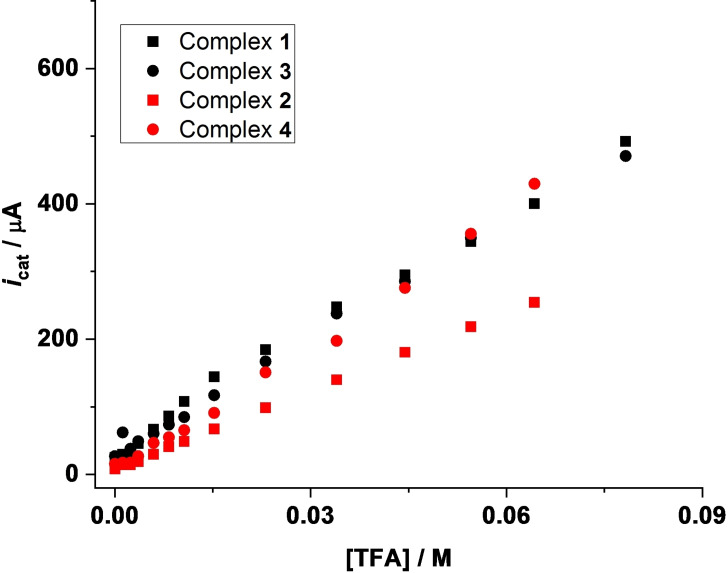
Plots of *i*_cat_/μA vs. [TFA]/m for the first reduction peak of **1** (▪), **3** (•), **2** (▪) and **4** (•) at a scan rate of 0.1 V s^−1^. The negative sign for the current has been ignored.

As can be seen from the current versus acid concentration plots, mono‐substituted complexes **1** and **3** display comparable catalytic currents (Figure 6). However, in the case of di‐substituted complexes, higher currents are observed for the bdt‐bridged complex **4** in comparison to the pdt‐bridged complex **2** (Figure 6).

In the presence of HClO_4_, complexes **2** and **4** showed higher currents than **1** and **3** as shown in Figure S17 (see Supporting Information). Furthermore, the observed higher currents for the aromatic (bdt) dithiolate complexes (**3** and **4**) are higher compared to the aliphatic (pdt) systems (**1** and **2**). Therefore, we can say that the bdt‐bridged complexes are better catalysts than the corresponding pdt‐bridged complexes. Strong acids (p*K*_a_ <10) are able to protonate the reduced intermediates more easily than weak acids. Moreover, due to the aromatic nature of the thiolate ligand in **3** and **4**, the one‐/two‐electron‐reduced species are easier to protonate in comparison to the intermediates formed from the pdt‐bridged complexes.

### Reaction Mechanism of Hydrogen Evolution Reaction

#### Proton Reduction by Aliphatic Di‐thiolate Complexes 1 and 2

**Scheme 2:**
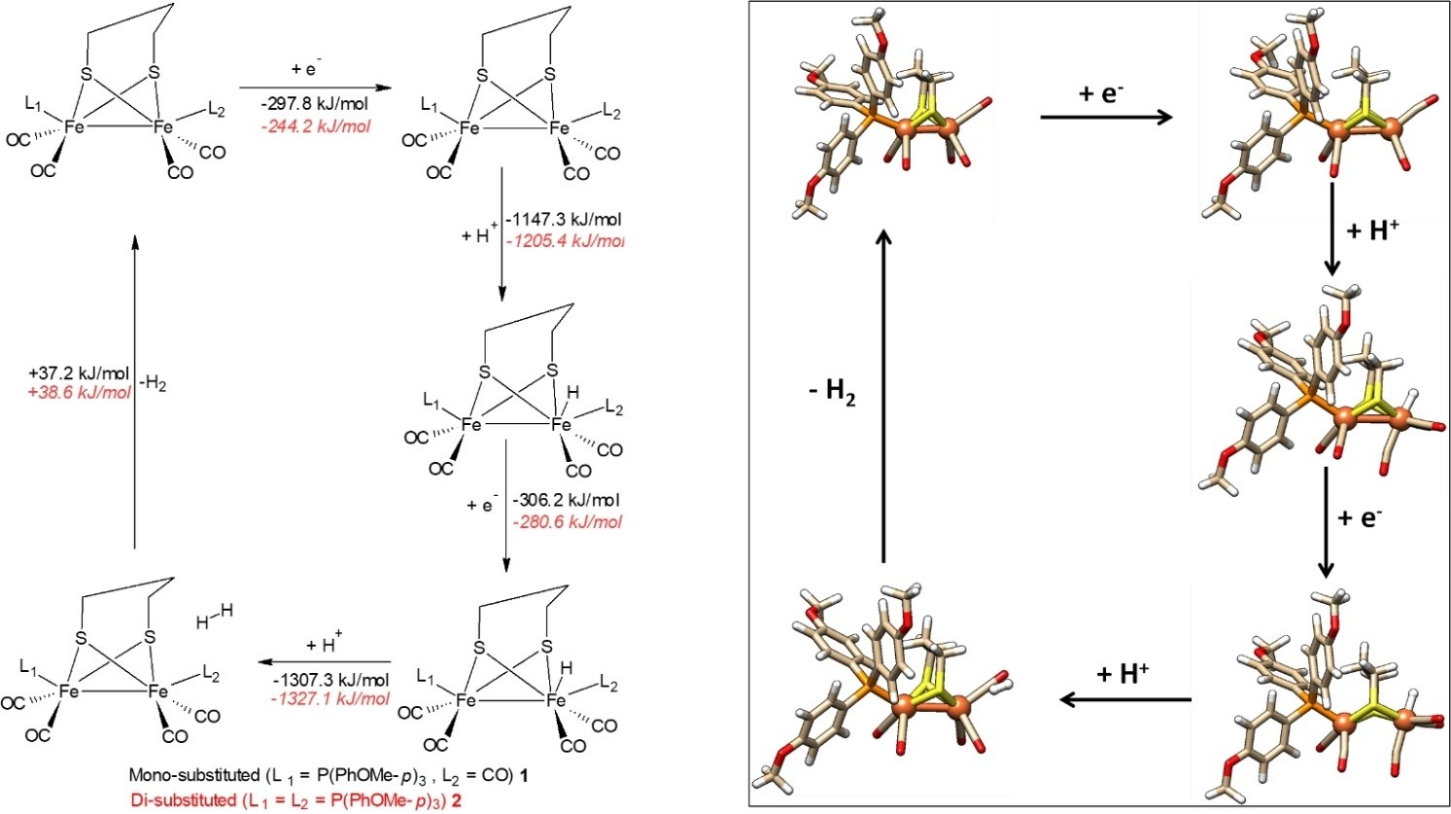
Calculated ECEC reaction mechanism for proton reduction by propane di‐thiolate complexes **1** (in black) and **2** (in red). Gibbs free energies in kJ mol^−1^ (BP86D3/def2‐TZVP(COSMO)). Hybrid DFT results and alternative pathways can be found in the Supporting Information.

The ECEC reaction mechanism for proton reduction of complexes **1** and **2** and calculated values of ΔG (in acetonitrile) are provided in Scheme 2. The optimized structures of each species in the ECEC reactions of complex **1** are given in Scheme 2 (on the right), while those for complex **2** are given in Scheme S1 (see Supporting Information). The changes in free energies (ΔG) calculated in the absence of solvent are given in Tables S8–S10 (see Supporting Information).

Upon the first one‐electron reduction, the unpaired spin density is delocalized over both iron atoms in **1^−^** and **2^−^**. This shows the redox activity of both iron atoms in the pdt‐bridged complexes, independent of the number of terminal phosphine ligands. In the mono‐reduced species, the complexes stay intact, and there is no free coordination site.

Following reduction (E), the first protonation step (C) leads to the formation of terminal iron hydride species **1 FeH** and **2 FeH** with Fe−H bond distances of 1.53 Å. Protonation is energetically feasible with negative values of ΔG for **1FeH** (−1147.3 kJ mol^−1^) and **2 FeH** (−1205.4 kJ mol^−1^). This shows that the terminal phosphine ligand(s) only slightly affect the proton affinity of iron. Protonation of a bridging thiolate is energetically less favourable (by 28–45 kJ mol^−1^) than of the Fe atom. In **1 FeH** and **2 FeH**, the geometry around the protonated Fe ion is distorted octahedral. The Fe−Fe bond length increases from 2.87 Å to 2.95 Å (in **1^−^** →**1 FeH**) and from 2.67 Å to 2.95 Å (in **2^−^** →**2 FeH**) while no significant changes are observed in the Fe−S bond distances. After the second one‐electron reduction step (E), the Fe−Fe bond distances further increase from 2.95 to 3.32 Å (**1 FeH** to **1 FeH^−^**) and from 2.95 to 3.33 Å (**2 FeH** to **2 FeH^−^**), while again no significant changes are observed in the Fe−S bond distances. The negative values of ΔG clearly show that the formation of **1 FeH^−^** (−306.2 kJ mol^−1^) as well as that of **2 FeH** ^−^ (−280.6 kJ mol^−1^) is thermodynamically feasible.

In the second protonation step (C), only the terminal hydride is available. When protonated, loosely bound complexes **1 ⋅ H_2_** and **2 ⋅ H_2_** are formed (with a H−H distance of 0.75 Å and a Fe⋅⋅⋅H_2_ distance of 4–6 Å). Moreover, in these species, the Fe−Fe distance decreases from 3.32 to 2.85 Å (**1FeH^−^** to **1 ⋅ H_2_**) and from 3.33 to 2.85 Å (**2FeH^−^** to **2 ⋅ H_2_**).

Protonation of one of the bridging S atoms is energetically less favorable (by 150–162 kJ mol^−1^) than at the terminal hydride atom. Stabilization of complexes **1 ⋅ H_2_** and **2 ⋅ H_2_** is weak and +37.2 kJ mol^−1^ (for **1 ⋅ H_2_** →**1**+**H_2_**) and +38.6 kJ mol^−1^ (for **2 ⋅ H_2_** →**2**+**H_2_**) of Gibbs free energy are needed for the dissociation of H_2_ from the complexes.

#### Proton Reduction by Aromatic Di‐thiolate Complexes 3 and 4

**Scheme 3:**
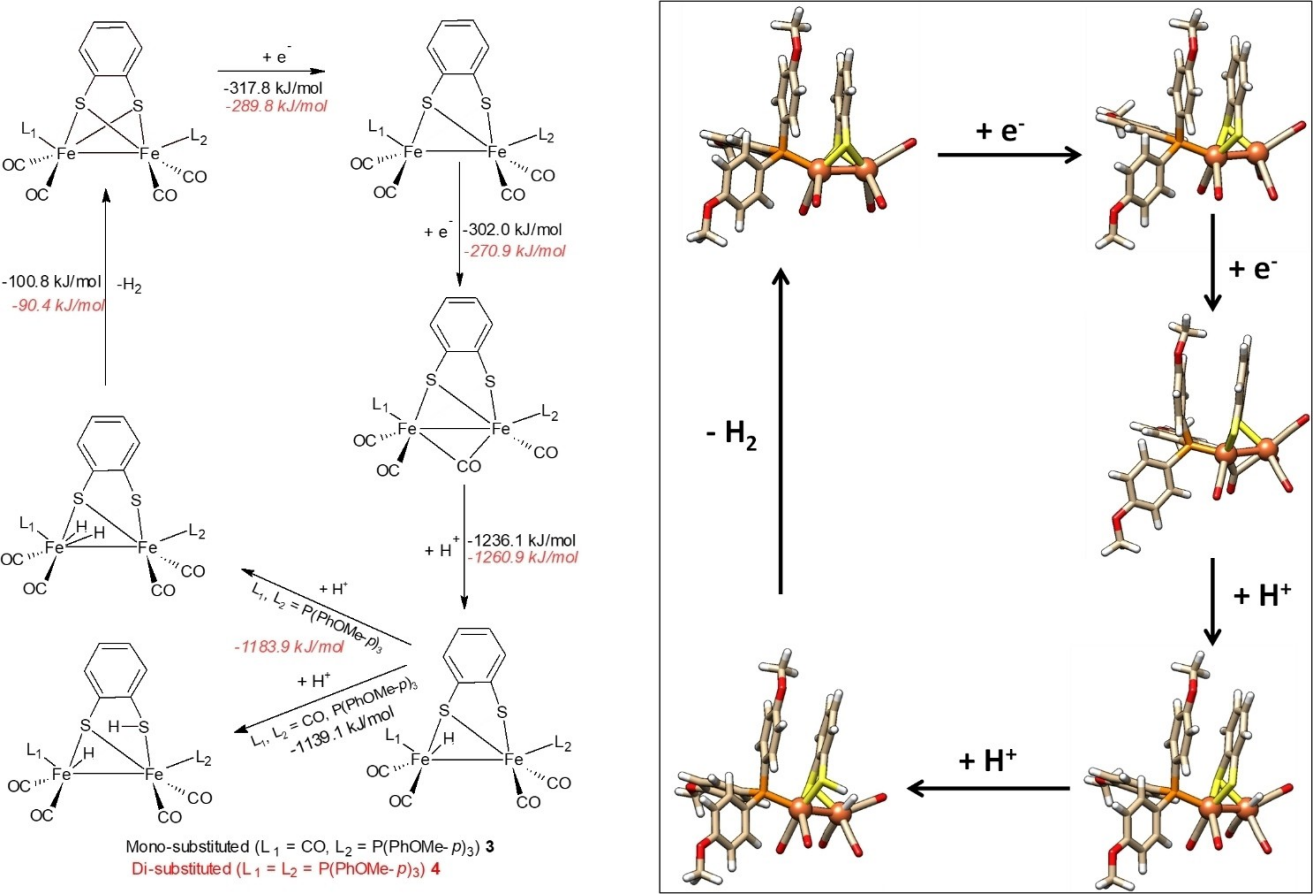
Calculated EECC reaction mechanism for proton reduction by benzene di‐thiolate complexes **3** (in black) and **4** (in red). Gibbs free energies in kJ mol^−1^ (BP86D3/def2‐TZVP(COSMO)). Hybrid DFT results and alternative pathways can be found in the Supporting Information.

For complexes **3** and **4**, the EECC reaction mechanism with calculated changes in ΔG in acetonitrile solvent are given in Scheme 3, while the alternative ECEC reaction mechanism is reported in Scheme S2. Changes in Gibbs free energies in the absence of solvent are given in Tables S11–S13 (see Supporting Information). The optimized structures of each species in the EECC and ECEC reaction mechanisms are provided in Scheme 3 (on the right) and in Schemes S3‐S5 (see Supporting Information). As discussed before, one of the Fe−S bonds breaks during the first reduction to give species **3^−^** and **4^−^**. In the second one‐electron reduction (E) step, in complexes **3^2−^** and **4^2−^** the terminal CO ligands re‐arrange and afford a μ‐bridging CO. The Fe−Fe bond distance only slightly elongates from 2.56 Å in **3^−^** to 2.69 Å in **3^2−^** Å and from 2.58 Å in **4^−^** to 2.76 Å in **4^2−^**. The Fe−S distances in the dissociated thiolate increase from 3.06 to 3.90 Å (**3^−^** to **3^2−^**) and from 3.60 Å to 3.94 Å (**4^−^** to **4^2−^**). The first chemical (C) step of protonating **3^2−^** and **4^2−^** can potentially occur either at the Fe atom or at the dissociated thiolate sulfur center. Protonation of the sulfur atom is not stable and shows an immediate intramolecular proton transfer from the S to the Fe atom (also observed in the ECEC mechanism, Scheme S2 in Supporting Information). Protonation at the Fe atom is, in contrast, found to be energetically favored and leads to the formation of terminal hydride species **3 FeH^−^** and **4 FeH^−^** with a Fe−H distance of 1.53 Å as also observed for complexes **1** and **2** (see above). Formation of **3 FeH^−^** and **4 FeH^−^** is not associated with changes in Fe−Fe bond distances, but the Fe⋅⋅⋅S distances of the dissociated thiolate decrease from 3.90 Å in **3^2−^** to 3.65 Å in **3 FeH^−^** and from 3.94 Å in **4^2−^** to 3.68 Å in **4 FeH^−^**. In these complexes, the bridging CO re‐orients and CO ligands become terminal again. The proton affinity is −1236.1 kJ mol^−1^ for **3 FeH^−^** and −1260.9 kJ mol^−1^ for **4 FeH^−^**, which shows a mild effect of phosphine ligand introduction. Finally, the second protonation of **3 FeH^−^** occurs at the bridging S atom to give **3 FeHSH** with a change in Gibbs free energy of −1139.1 kJ mol^−1^. In complex **4 FeH^−^**, however, the second protonation occurs at the terminal iron hydride, which leads to the formation of **4 FeH_2_**. Here, the calculated proton affinity of −1183.9 kJ mol^−1^ of **4 FeH^−^** is larger by 45 kJ mol^−1^ compared to **3 FeH^−^**. The H−H distances amount to 1.49 Å and 0.90 Å in **3 FeHSH** and **4 FeH_2_**, respectively. The Fe−Fe bond lengths are 2.69 Å and 2.68 Å, while Fe⋅⋅⋅S distances are 3.56 Å and 3.71 Å in **3 FeHSH** and **4 FeH_2_**, respectively.

Finally, from **3 FeHSH** and **4 FeH_2_**, we can recover complexes **3** and **4** by releasing molecular hydrogen. This step is exothermic with ΔG values of −100.8 kJ mol^−1^ and −90.4 kJ mol^−1^ for **3 FeHSH** and **4 FeH_2_**, respectively.

## Conclusions

Homo di‐nuclear propane di‐thiolate bridged complexes **1** and **2** and benzene di‐thiolate bridged complexes **3** and **4** with either one or two terminal phosphine ligands display catalytic activity towards proton reduction and hydrogen evolution. The electron‐donating substituents on the ligand lead to an increased electron density at the iron centers, resulting in a higher catalytic current in di‐ compared to mono‐substituted complexes. The structural parameters, IR spectra and electrochemical properties of complexes **1**–**4**, calculated using the quantum chemical method, are in good agreement with the experimental data.

The complexes were tested as electrocatalysts for HER in the presence of three different acids. The first one‐electron reduction potential of the aliphatic pdt‐bridged complexes **1** (−1.87 V) and **2** (−2.10 V) is found to be higher than that of the aromatic bdt‐bridged complexes **3** (−1.65 V) and **4** (−2.02 V). Moreover, we observed a higher current for the aromatic dithiolate complexes (**3** and **4**) in comparison to the aliphatic systems (**1** and **2**). DFT calculations demonstrate that the reaction mechanisms for aromatic and aliphatic bridged complexes are strikingly different.

On one‐electron reduction in the bdt‐bridged complexes, one of the Fe−S bonds breaks while all bonds remain intact in the pdt‐bridged complexes. Therefore, the bdt‐bridged reduced species **3^−^** and **4^−^** possess an accessible site for protonation while no free coordination site is available in the pdt‐bridged reduced species **1^−^** and **2^−^**. Hydrogen release appears to be kinetically facile for pdt‐bridged (**1** and **2**) complexes and thermodynamically feasible in the bdt‐bridged complexes (**3** and **4**). Therefore, the bdt‐bridged complexes **3** and **4** are labelled as displaying a superior catalytic behavior than the pdt‐bridged ones. An EECC reaction mechanism for hydrogen reduction for the bdt‐bridged complexes (**3** and **4**) is most plausible, since first and second redox potentials are very close and occur at lower potentials. For the pdt‐bridged complexes (**1** and **2**), the second one‐electron reduction occurs at a higher potential and, therefore, an ECEC reaction mechanism for the pdt‐bridged complexes appears more plausible.

## Experimental Section

### Materials and Measurements

All experiments were carried out under an inert atmosphere using Schlenk line techniques. The reagents (Fe)_3_(CO)_12_, 1,2‐benzenedithiolate, 1,3‐propanedithiolate and tris(4‐methoxyphenyl)phosphine were purchased from Sigma‐Aldrich and used as received. The all‐carbonyl precursor complexes [Fe_2_(μ‐bdt)(CO)_6_] and [Fe_2_(μ‐pdt)(CO)_6_] were prepared according to reported procedures.[^41^, ^42^] All anhydrous solvents (dichloromethane, acetonitrile, toluene) were obtained from Sigma‐Aldrich and used without further purification. Deuterated solvents were also obtained from Sigma‐Aldrich. The ^1^H and ^31^P NMR spectra were recorded at room temperature in CDCl_3_ solution with a JEOL 400 MHz NMR Spectrometer. FTIR spectra were recorded from dichloromethane (CH_2_Cl_2_) solutions of the samples over the range 400–4000 cm^−1^ on a Perkin Elmer FTIR Spectrometer. Elemental (C, H and N) analyses were performed on a Vario Micro Cube elemental analyzer.

### X‐Ray Crystallography

Single crystals of **2**, **3** and **4** were grown by slow evaporation of hexane/dichloromethane solutions at low temperature. X‐ray data for **2**–**4** were collected on Oxford X‐Calibur‐S single crystal X‐ray diffractometer using Mo‐Kα radiation. Significant crystallographic parameters and refinement details are listed in Tables S1–S3 (Supporting Information). The structures were solved and refined by full‐matrix least‐squares techniques on F2 using SHELX‐97 (SHELXTL program package).[^64^, ^65^, ^66^] For the molecular graphics, the program OLEX^2^ was used.^[67]^ Deposition Number(s) (for 2), 2111654 (for 3), 2111656 (for 4) (for **2**), 2111654 (for **3**), 2111656 (for **4**) contain the supplementary crystallographic data for this paper. These data are provided free of charge by the joint Cambridge Crystallographic Data Centre and Fachinformationszentrum Karlsruhe.

### Electrochemistry

Electrochemical measurements were conducted in acetonitrile with 0.1 m tetrabutylammonium hexafluorophosphate (Fluka, electrochemical grade) as supporting electrolyte that was dried in vacuo at 383 K. Cyclic voltammetry was carried out using an Autolab potentiostat with a GPES/Nova 1.6 electrochemical interface. For cyclic voltammetry, the working electrode was a glassy carbon disc (diameter 3 mm, freshly polished). A platinum wire was used as counter electrode and the reference electrode was a non‐aqueous Ag/Ag^+^ electrode (CH Instruments, 0.010 m AgNO_3_ in acetonitrile). All the potentials (text, tables, and figures) are quoted against the ferrocene‐ferrocenium couple (Fc/Fc^+^); ferrocene was added as an internal standard at the end of the experiments. All solutions were prepared from dry dichloromethane and acetonitrile (Sigma‐Aldrich, spectroscopic grade, dried with MS 3 Å).

### Computational Details

All the DFT calculations were carried out with Turbomole 7.2^[68]^ and Grimme's D3 dispersion‐corrected^[69]^ B3LYP and BP86 exchange‐correlation functional[^70^, ^71^, ^72^, ^73^] and def2‐TZVP basis set.[^74^, ^75^] The resolution‐of‐identity (RI) approximation[^76^, ^77^] has been used in all calculations. The complexes are optimized in acetonitrile solvent (dielectric constant (ε)=37.5) using the COSMO solvation model.[^78^, ^79^] All solvent‐optimized structures were further optimized in vacuum. To validate the minimum energy structure as well as to calculate thermodynamic properties, we have performed the frequencies calculation on the gas‐phase‐optimized structures at the same level of theory. Redox potentials were calculated following the references[^80^, ^81^] and the redox potential of all complexes is reported relative to Fc/Fc^+^ reference electrode in acetonitrile.

## Conflict of interest

The authors declare no conflict of interest.

1

## Supporting information

As a service to our authors and readers, this journal provides supporting information supplied by the authors. Such materials are peer reviewed and may be re‐organized for online delivery, but are not copy‐edited or typeset. Technical support issues arising from supporting information (other than missing files) should be addressed to the authors.

Supporting Information

## Data Availability

The data that support the findings of this study are available in the supplementary material of this article.
