# Supplementary material for: Mechanism of Diiron Hydrogenase Complexes Controlled by Nature of Bridging Dithiolate Ligand
Source: ChemistryOpen. 2022 Jan 4;11(1):e202100238. doi: 10.1002/open.202100238 (PMC8734113; doi:10.1002/open.202100238)
Supplement: Supplementary file 1 — Supporting Information [file OPEN-11-e202100238-s001.pdf]

ChemistryOpen

Supporting Information

Mechanism of Diiron Hydrogenase Complexes Controlled by Nature of Bridging Dithiolate Ligand

Mookan Natarajan, Naveen Kumar, Meenakshi Joshi, Matthias Stein,* and Sandeep Kaur-Ghumaan*

Synthesis and Characterization of [Fe_2(μ-pdt)$(CO)_4$(P(PhOMe-*p*)$_3$)$_2$] 2

Complex **1** was synthesized as reported in literature. A mixture of [Fe_2(μ-pdt)$(CO)_6$] (200 mg, 0.52 mmol) and Tris(4-methoxyphenyl)phosphine (733 mg, 2.08 mmol) were dissolved in toluene and refluxed for 3 h under argon atmosphere. The resulting solution was evaporated to dryness under vacuum and the residue was chromatographed on a silica gel column. Elution with a mixture of hexane/ dichloromethane (1:1, 2:3 v/v) afforded orange-red and red solutions of a **1** and **2**. Removal of solvent under vacuum resulted in red products for both the complexes. Complex **2** was recrystallized from hexane and dichloromethane solution at -4˚ C.

For **2**: Yield: 0.011 g (2 %). ^{1}H NMR (400 MHz, $CDCl_3$): δ = 6.85-6.48 (m, 12H), 3.75 (s, 9H), 2.09 (s, 4H), 1.16 (s, 2H) ppm; ^{31}P NMR (161.8 MHz, $CDCl_3$): δ = 41.5 (s) ppm; FTIR (CH_2Cl_2): ῡ = 1988, 1944, 1923, 1897 cm^{-1} (C=O); elemental analysis calcd (%) for $C_{49}H_{48}Fe_2O_{10}P_2S_2$: C 56.88, H 4.68; found: C 56.78, H 4.59.

Synthesis of and Characterization of [Fe_2(μ-bdt)$(CO)_5$(P(PhOMe-*p*)$_3$)] 3 and [Fe_2(μ-bdt)$(CO)_4$(P(PhOMe-*p*)$_3$)$_2$] 4

A mixture of [Fe_2(μ-bdt)$(CO)_6$] (240 mg, 0.56 mmol) and Tris(4-methoxyphenyl)phosphine (789 mg, 2.24 mmol) were dissolved in toluene and refluxed for 4 h under argon atmosphere. The resulting solution was evaporated to dryness under vaccum and the residue was chromatographed on a silica gel column. Elution with a mixture of hexane/dichloromethane (1:1, 2:3 v/v) afforded orange-red and dark red solutions of **3** and **4**. Complex **3** was obtained as an air-stable orange-red powder whereas complex **4** was obtained as a dark red solid on evaporation of the solvent. **3** and **4** were then recrystallized from a hexane/CH_2Cl_2 mixture at at -4 ^{o}C.

For **3**: Yield: 0.220 g (41 %). ^{1}H NMR (400 MHz, $CDCl_3$): δ = 7.38-6.81 (m, 12H), 6.49 (m, 2H), 6.19 (m, 2H), 3.80 (s, 9H) ppm; ^{31}P NMR (161.8 MHz, $CDCl_3$): 58.2 (s) ppm; FTIR (CH_2Cl_2): ῡ = 2045, 1984, 1961, 1932 cm^{-1} (C=O); elemental analysis calcd (%) for $C_{32}H_{25}Fe_2S_2PO_8$: C 51.64, H 3.63; found: C 51.42, H 3.39.

For **4**: Yield: 0.060 g (8 %). ^{1}H NMR (400 MHz, $CDCl_3$): δ = 7.29-6.69 (m, 24H), 5.85 (m, 2H), 5.68 (m, 2H), 3.69 (s, 9H) ppm; ^{31}P NMR (161.8 MHz, $CDCl_3$): 35.6 (s) ppm; FTIR (CH_2Cl_2): ῡ = 1996, 1953, 1932, 1909 cm^{-1} (C=O); elemental analysis calcd (%) for $C_{52}H_{46}Fe_2S_2P_2O_{10}$: C 58.44, H 4.34; found: C 58.42, H 4.39.

Table S1. Crystallographic data for complexes **2**, **3** and **4**.

	**2**	**3**	**4**
empirical formula	$C_{49}H_{48}Fe_2O_{10}P_2S_2$	$C_{32}H_{25}Fe_2O_8PS_2$	$C_{52}H_{46}Fe_2O_{10}P_2S_2$
formula weight	1034.63	744.31	1068.65
crystal system	triclinic	monoclinic	triclinic
space group	P-1	P 1 2_1/c 1	P-1
a, Å	13.0586(3)	10.3450(4)	10.1705(15)
b, Å	14.0818(3)	18.7838(6)	12.1052(17)
c, Å	14.5927(3)	17.2205(6)	23.473(3)
α, deg	78.480(2)	90.00	90.066(11)
β, deg	76.373(2)	97.519(4)	102.071(12)
γ, deg	76.075(2)	90.00	96.043(12)
V, Å^3	2501.99(9)	3317.5(2)	2809.5(7)
Z	2	4	2
D_{calc}, g.cm^{-3}	1.373	1.490	1.263
T, Kelvin	298(2)	293(2)	293(2)
crystal size, mm	0.33 x 0.27 x 0.24	0.11 x 0.11 x 0.11	0.26 x 0.25 x 0.24
μ, mm^{-1}	0.782	1.097	0.699
no. of reflns meads	33127	32034	21341
no. of unique reflns	10213	6772	9798
no. of reflns obsd.	8063 [$I > 2\sigma(I)$]	5957 [$I > 2\sigma(I)$]	5003 [$I > 2\sigma(I)$]
no. of parameters	592	450	619
R_1 / wR$_2$ [$I > 2\sigma(I)$]	0.0509/0.1265	0.0417/0.0879	0.0831/0.1176
R_1 / wR_2 [all refl.]	0.0667/0.1357	0.0492/0.0911	0.1563/0.1404
goodness of fit	1.032	1.144	0.943
$\Delta\rho$, e.Å^{-3}	1.692/-0.567	0.375/-0.242	0.477/-0.452

Table S2. Selected bond lengths [in Å] and angles [in °] for **2** and **3** observed from X-ray diffraction.

**2**				**3**			
Bond lengths		Bond angles		Bond lengths		Bond angles	
Fe(2)-S(1)	2.2675(9)	S(1)-Fe(2)-S(2)	83.58(3)	Fe(1)-S(1)	2.2755(8)	S(2)-Fe(1)-S(1)	80.48(3)
Fe(2)-S(2)	2.2722(9)	S(1)-Fe(2)-Fe(1)	56.32(3)	Fe(1)-S(2)	2.2645(8)	S(1)-Fe(2)-Fe(1)	56.51(2)
Fe(1)-S(1)	2.2756(10)	S(2)-Fe(2)-Fe(1)	56.04(2)	Fe(2)-S(1)	2.2949(8)	S(2)-Fe(2)-Fe(1)	56.32(2)
Fe(1)-S(2)	2.2673(9)	S(2)-Fe(1)-S(1)	83.51(3)	Fe(2)-S(2)	2.2848(8)	S(2)-Fe(2)-S(1)	79.64(3)
Fe(1)-Fe(2)	2.5296(6)	S(1)-Fe(1)-Fe(2)	56.01(3)	Fe(1)-Fe(2)	2.4971(5)	S(1)-Fe(1)-Fe(2)	57.26(2)
Fe(2)-P(2)	2.2542(9)	S(2)-Fe(1)-Fe(2)	56.23(2)	Fe(2)-P(1)	2.2580(7)	S(2)-Fe(1)-Fe(2)	57.10(2)
Fe(1)-P(1)	2.2456(9)	Fe(2)-S(1)-Fe(1)	67.67(3)	Fe(2)-C(1)	1.771(3)	Fe(1)-S(1)-Fe(2)	66.23(2)
Fe(2)-C(4)	1.760(4)	Fe(1)-S(2)-Fe(2)	67.73(3)	Fe(2)-C(2)	1.765(3)	Fe(1)-S(2)-Fe(2)	66.58(2)
Fe(2)-C(3)	1.764(4)	C(4)-Fe(2)-P(2)	95.37(13)	Fe(1)-C(3)	1.782(4)	C(2)-Fe(2)-P(1)	96.22(10)
Fe(1)-C(2)	1.759(4)	C(3)-Fe(2)-P(2)	96.61(12)	Fe(1)-C(4)	1.778(3)	C(1)-Fe(2)-P(1)	94.85(10)
Fe(1)-C(1)	1.754(4)	C(4)-Fe(2)-C(3)	93.78(17)	Fe(1)-C(5)	1.808(4)	C(2)-Fe(2)-C(1)	91.78(14)
O(4)-C(4)	1.151(4)	C(1)-Fe(1)-C(2)	91.4(2)	O(1)-C(1)	1.139(4)	P(1)-Fe(2)-Fe(1)	152.60(3)
O(3)-C(3)	1.142(4)	C(2)-Fe(1)-P(1)	95.52(13)	O(2)-C(2)	1.139(4)	C(5)-Fe(1)-Fe(2)	151.48(10)
O(2)-C(2)	1.147(5)	C(1)-Fe(1)-P(1)	95.70(13)	O(3)-C(3)	1.142(4)		
O(1)-C(1)	1.146(5)	C(1)-Fe(1)-Fe(2)	102.89(13)	O(4)-C(4)	1.136(4)		
		P(1)-Fe(1)-Fe(2)	154.93(3)	O(5)-C(5)	1.122(4)		
		P(2)-Fe(2)-Fe(1)	156.11(3)				

Table S3. Selected bond lengths (in Å) and angles (in °) for complex **4** from X-ray diffraction.

Bond lengths		Bond angles			
Fe(2)-S(1)	2.2984(17)	S(1)-Fe(2)-S(2)	79.58(6)	P(1)-Fe(2)-S(1)	104.07(6)
Fe(2)-S(2)	2.2796(19)	S(1)-Fe(2)-Fe(1)	57.00(5)	C(2)-Fe(2)-S(2)	90.5(2)
Fe(1)-S(1)	2.2991(17)	S(2)-Fe(2)-Fe(1)	56.78(5)	C(1)-Fe(2)-S(2)	156.1(2)
Fe(1)-S(2)	2.2838(17)	S(1)-Fe(1)-S(2)	79.48(6)	P(1)-Fe(2)-S(2)	103.91(7)
Fe(1)-Fe(2)	2.5052(13)	S(1)-Fe(1)-Fe(2)	56.97(5)	C(2)-Fe(2)-Fe(1)	100.4(2)
Fe(2)-P(1)	2.2496(19)	S(2)-Fe(1)-Fe(2)	56.62(5)	C(1)-Fe(2)-Fe(1)	99.5(2)
Fe(1)-P(2)	2.2517(19)	Fe(2)-S(1)-Fe(1)	66.04(5)	P(1)-Fe(2)-Fe(1)	153.03(6)
Fe(2)-C(2)	1.765(6)	Fe(2)-S(2)-Fe(1)	66.59(5)	C(4)-Fe(1)-S(1)	154.9(2)
Fe(2)-C(1)	1.757(7)	C(2)-Fe(2)-P(1)	98.3(2)	C(3)-Fe(1)-(S1)	88.9(2)
Fe(1)-C(4)	1.770(6)	C(1)-Fe(2)-P(1)	99.3(2)	P(2)-Fe(1)-S(1)	109.17(7)
Fe(1)-C(3)	1.724(7)	C(2)-Fe(2)-C(1)	91.4(3)	C(4)-Fe(1)-S(2)	91.99(19)

O(1)-C(1)	1.140(7)	C(4)-Fe(1)-C(3)	92.2(3)	C(3)-Fe(1)-S(2)	160.9(2)
O(2)-C(2)	1.159(6)	C(3)-Fe(1)-P(2)	96.8(2)	P(2)-Fe(1)-S(2)	101.29(7)
O(4)-C(4)	1.160(6)	C(4)-Fe(1)-P(2)	95.6(2)	C(4)-Fe(1)-Fe(2)	98.63(19)
O(3)-C(3)	1.157(6)	C(2)-Fe(2)-S(1)	157.1(2)	C(3)-Fe(1)-Fe(2)	104.3(2)
		C(1)-Fe(2)-S(1)	89.8(2)	P(2)-Fe(1)-Fe(2)	153.93(6)

Table S4. Selected bond lengths [in Å] and angles [in °] for **2** and **3** calculated using BP86-D3/def2-TZVP ([a]B3LYP-D3/def2-TZVP).

2				3			
Bond lengths		Bond angles		Bond lengths		Bond angles	
Fe(1)-S(1)	2.28 (2.31)	S(1)-Fe(1)-S(2)	83.79 (83.83)	Fe(1)-S(1)	2.31 2.33)	S(2)-Fe(1)-S(1)	79.75 (79.74)
Fe(1)-S(2)	2.30 (2.32)	S(1)-Fe(1)-Fe(2)	56.52 (57.13)	Fe(1)-S(2)	2.30 (2.33)	S(1)-Fe(2)-Fe(1)	57.13 (57.56)
Fe(2)-S(1)	2.29 (2.32)	S(2)-Fe(1)-Fe(2)	56.66 (57.23)	Fe(2)-S(1)	2.30 (2.34)	S(2)-Fe(2)-Fe(1)	56.92 (57.38)
Fe(2)-S(2)	2.30 (2.33)	S(2)-Fe(2)-S(1)	83.47 (83.44)	Fe(2)-S(2)	2.30 (2.33)	S(2)-Fe(2)-S(1)	79.94 (79.63)
Fe(1)-Fe(2)	2.54 (2.53)	S(1)-Fe(2)-Fe(1)	56.20 (56.73)	Fe(1)-Fe(2)	2.51 (2.50)	S(1)-Fe(1)-Fe(2)	56.87 (57.60)
Fe(1)-P(2)	2.22 (2.26)	S(2)-Fe(2)-Fe(1)	56.44 (56.97)	Fe(2)-P(1)	2.23 (2.27)	S(2)-Fe(1)-Fe(2)	56.82 (57.55)
Fe(2)-P(1)	2.23 (2.27)	Fe(1)-S(1)-Fe(2)	67.28 (66.13)	Fe(2)-C(21)	1.76 (1.78)	Fe(1)-S(1)-Fe(2)	66.00 (64.84)
Fe(1)-C(19)	1.76 (1.77)	Fe(2)-S(2)-Fe(1)	66.91 (65.80)	Fe(2)-C(22)	1.76 (1.78)	Fe(1)-S(2)-Fe(2)	66.26 (65.07)
Fe(1)-C(22)	1.76 (1.78)	C(19)-Fe(1)-P(2)	95.86 (96.67)	Fe(1)-C(19)	1.78 (1.80)	C(22)-Fe(2)-P(1)	96.82 (97.17)
Fe(2)-C(20)	1.75 (1.77)	C(22)-Fe(1)-P(2)	96.64 (96.96)	Fe(1)-C(20)	1.79 (1.81)	C(21)-Fe(2)-P(1)	96.78 (97.17)
Fe(2)-C(21)	1.76 (1.78)	C(19)-Fe(1)-C(22)	91.20 (92.30)	Fe(1)-C(23)	1.77 (1.80)	C(22)-Fe(2)-C(21)	91.16 (92.00)
O(1)-C(19)	1.16 (1.15)	C(20)-Fe(2)-C(21)	91.59 (92.60)	O(1)-C(19)	1.16 (1.14)	P(1)-Fe(2)-Fe(1)	152.30 (152.78)
O(4)-C(21)	1.16 (1.15)	C(20)-Fe(2)-P(1)	94.76 (95.46)	O(2)-C(20)	1.16 (1.14)	C(20)-Fe(1)-Fe(2)	150.71 (149.94)
O(2)-C(20)	1.16 (1.15)	C(21)-Fe(2)-P(1)	94.29 (94.71)	O(5)-C(23)	1.16 (1.14)		
O(3)-C(21)	1.16 (1.15)	P(2)-Fe(1)-Fe(2)	154.47 (154.59)	O(3)-C(21)	1.16 (1.15)		
		P(1)-Fe(2)-Fe(1)	158.79 (158.69)	O(4)-C(22)	1.16 (1.14)		

Table S5. Selected bond lengths (in Å) and angles (in °) for complex **4** calculated using BP86-D3/def2-TZVP ([a]B3LYP-D3/def2-TZVP).

Bond lengths		Bond angles			
Fe(1)-S(1)	2.28 (2.31)	S(1)-Fe(1)-S(2)	83.79 (83.83)	P(2)-Fe(1)-S(1)	106.17 (105.80)
Fe(1)-S(2)	2.30 (2.32)	S(1)-Fe(1)-Fe(2)	56.52 (57.13)	C(22)-Fe(1)-S(2)	87.06 (86.63)
Fe(2)-S(1)	2.29 (2.32)	S(2)-Fe(1)-Fe(2)	56.66 (57.23)	C(19)-Fe(1)-S(2)	157.66 (88.70)
Fe(2)-S(2)	2.30 (2.33)	S(1)-Fe(2)-S(2)	83.47 (83.44)	P(2)-Fe(1)-S(2)	106.47 (105.62)
Fe(1)-Fe(2)	2.54 (2.53)	S(1)-Fe(2)-Fe(1)	56.20 (56.73)	C(22)-Fe(1)-Fe(2)	101.00 (100.19)
Fe(1)-P(2)	2.22 (2.25)	S(2)-Fe(2)-Fe(1)	56.44 (56.97)	C(19)-Fe(1)-Fe(2)	102.04 (101.20)
Fe(2)-P(1)	2.23 (2.27)	Fe(1)-S(1)-Fe(2)	67.28 (66.13)	P(2)-Fe(1)-Fe(2)	154.47 (154.59)
Fe(1)-C(19)	1.76 (1.77)	Fe(1)-S(2)-Fe(2)	66.90 (65.80)	C(20)-Fe(2)-S(1)	156.75 (156.72)
Fe(1)-C(22)	1.76 (1.78)	C(19)-Fe(1)-P(2)	95.86 (96.67)	C(21)-Fe(2)-S(1)	87.67 (87.20)
Fe(2)-C(20)	1.75 (1.77)	C(22)-Fe(1)-P(2)	96.64 (96.96)	P(1)-Fe(2)-S(1)	108.47 (107.76)
Fe(2)-C(21)	1.76 (1.78)	C(19)-Fe(1)-C(22)	91.20 (92.30)	C(20)-Fe(2)-S(2)	87.46 (87.09)
O(1)-C(19)	1.17 (1.15)	C(20)-Fe(2)-C(21)	91.59 (92.60)	C(21)-Fe(2)-S(2)	154.74 (154.95)
O(4)-C(22)	1.16 (1.15)	C(20)-Fe(2)-P(1)	94.76(95.46)	P(1)-Fe(2)-S(2)	110.95 (110.26)
O(2)-C(20)	1.1644 (1.15)	C(21)-Fe(2)-P(1)	94.29 (94.71)	C(20)-Fe(2)-Fe(1)	101.13 (100.42)
O(3)-C(21)	1.1620 (1.16)	C(22)-Fe(1)-(S1)	157.01 (156.94)	C(21)-Fe(2)-Fe(1)	99.18 (98.64)
		C(19)-Fe(1)-S(1)	89.35 (88.70)	P(1)-Fe(2)-Fe(1)	158.79 (158.70)

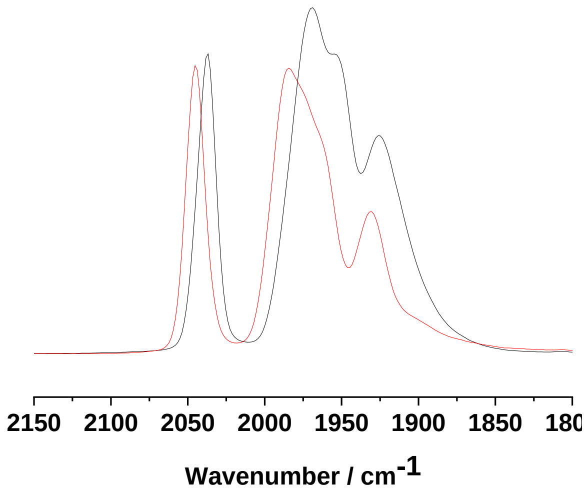

Figure S1. FTIR spectra of complexes **1** (——) and **3** (——) in dichloromethane.

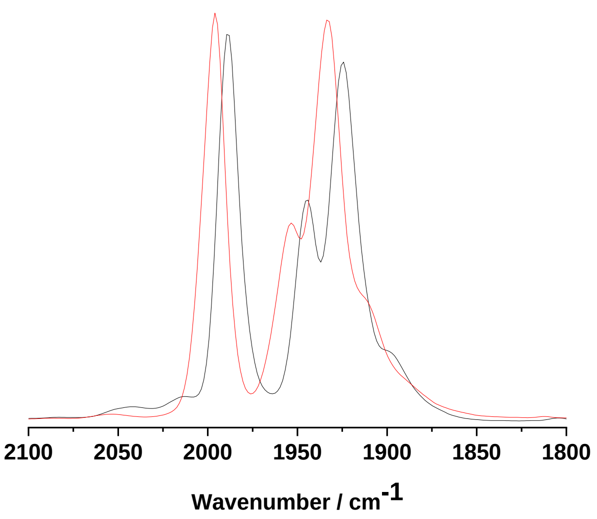

Figure S2. FTIR spectra of complexes **2** (——) and **4** (——) in dichloromethane.

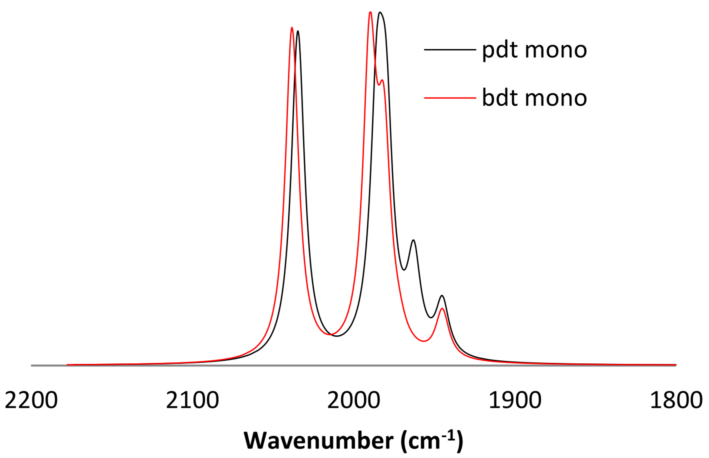

Figure S3. Calculated IR spectra of complexes **1** (——) and **3** (——) (BP86-D3/def2-TZVP).

Table S6. FTIR data for complexes **1-4** in dichloromethane.

Complex	Wavenumber [cm^{-1}]
**1**	2037, 1968, 1952, 1926
**3**	2045, 1984, 1961, 1932
**2**	1988, 1944, 1923, 1897
**4**	1996, 1953, 1932, 1909
[Fe_2(μ-pdt)$(CO)_6$]^a	2074 (m), 2036 (vs), 1995 (s)
[Fe_2(μ-bdt)$(CO)_6$]^b	2079 (m), 2044 (s), 2006 (vs), 1967 (vw), 1958 (vw)

^{a}acetonitrile, ^{b}hexane

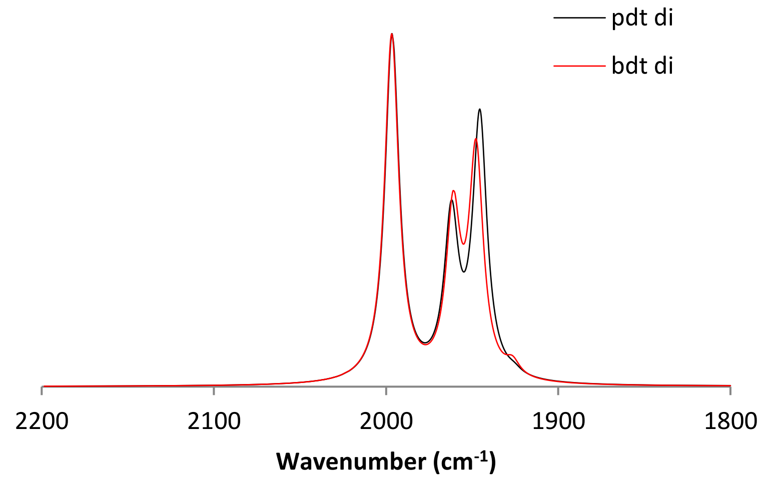

Figure S4. Calculated IR spectra of complexes **2** (——) and **4** (——) (B86-D3/def2-TZVP).

^{1}H and ^{31}P NMR Spectroscopy

The ^{1}H NMR spectrum of **2** in $CDCl_3$ displayed two multiplets at 6.85 and 6.48 ppm. In addition, there are two multiplets in the range of 1.6-1.4 ppm for the protons of the pdt ligand. On the other hand, the ^{1}H NMR spectrum of **3** in $CDCl_3$ (Figure S5) displayed two multiplets at 6.81 and 7.38 ppm. The two multiplets in the complexes **2** and **3** are due to the protons of the phenyl rings of the phosphine ligand, P(PhOMe-*p*)$_3$. The multiplets at 6.19 and 6.49 ppm for **3** appear from the protons of the bdt ligand. In addition, a singlet is observed at 3.75 and 3.80 ppm for **2** and **3**, respectively, for the methyl protons of the three methoxy groups. The proton signals for the all carbonyl bdt precursor complex appeared at 7.13 and 6.63 ppm.[1] In the ^{1}H NMR spectrum of complex **4**, multiplets were observed at 5.68, 5.85, 6.70 and 7.29 ppm in addition to the singlet at 3.69 ppm. The ^{31}P {^{1}H} NMR spectra for complexes **2**, **3** and **4** feature a singlet

at 41.48, 58.19 and 35.55 ppm, respectively (Figures S6-S8). For **1** the ^{31}P {^{1}H} signal has been reported as a singlet at 60.26 ppm.[2] The appearance of one peak in the phosphorus NMR of the di-substituted complexes suggests that the phosphine ligand on both the Fe centres has similar environment and that the complexes are symmetrical. The lower chemical shift values for **2** and **4** in comparison to **1** and **3**, respectively can be explained by the presence of two phosphine ligands, one on each of the iron centres.

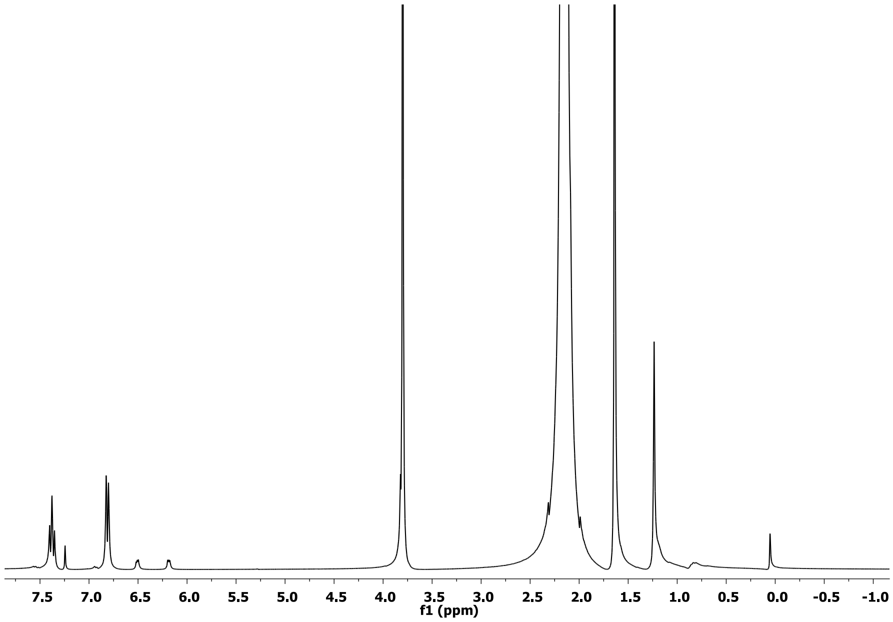

Figure S5. ^{1}H NMR spectrum of complex **3** in $CDCl_3$.

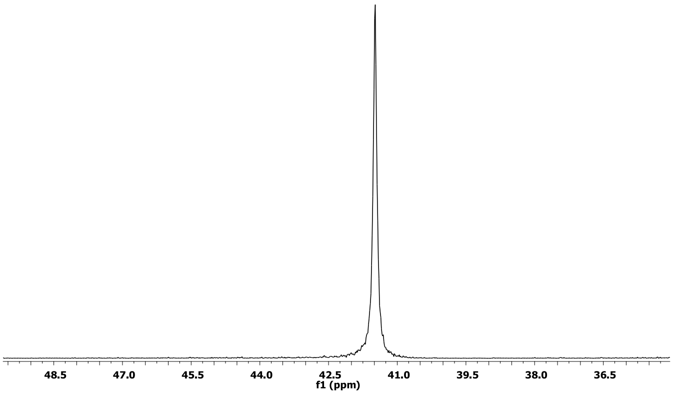

Figure S6. ^{31}P {^{1}H} NMR spectrum of complex **2** in $CDCl_3$.

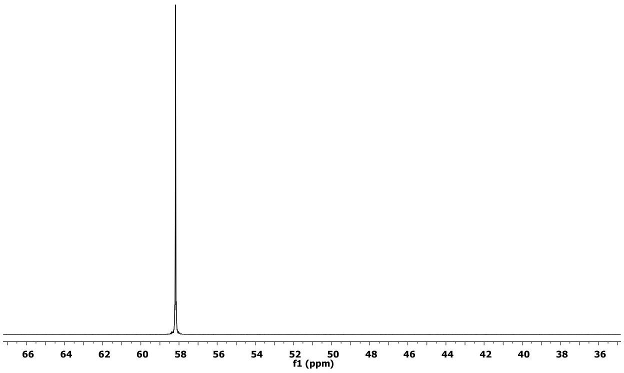

Figure S7. ^{31}P {^{1}H} NMR spectrum of complex **3** in $CDCl_3$.

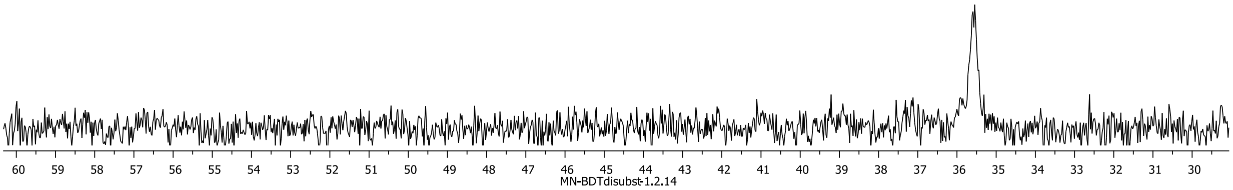

Figure S8. ^{31}P {^{1}H} NMR spectrum of complex **4** in $CDCl_3$.

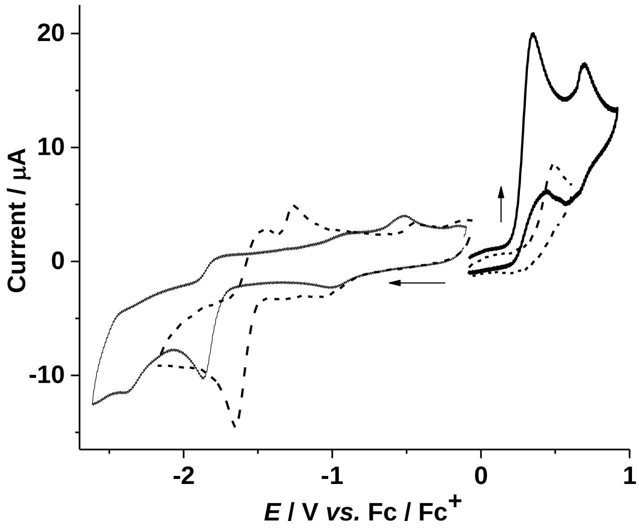

Figure S9. Cyclic voltammograms for mono-substituted complexes **1** (pdt) (—) and **3** (bdt) (----) in acetonitrile at a scan rate of 0.1 Vs^{-1}.

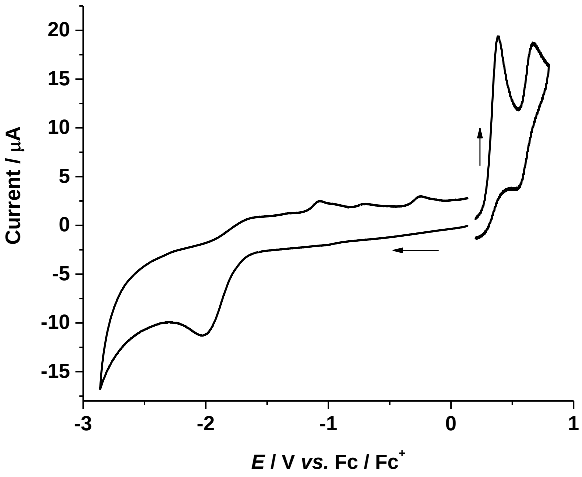

Figure S10. Cyclic voltammograms for complex di-substituted bdt complex **4** (----) in acetonitrile at a scan rate of 0.1 Vs^{-1}.

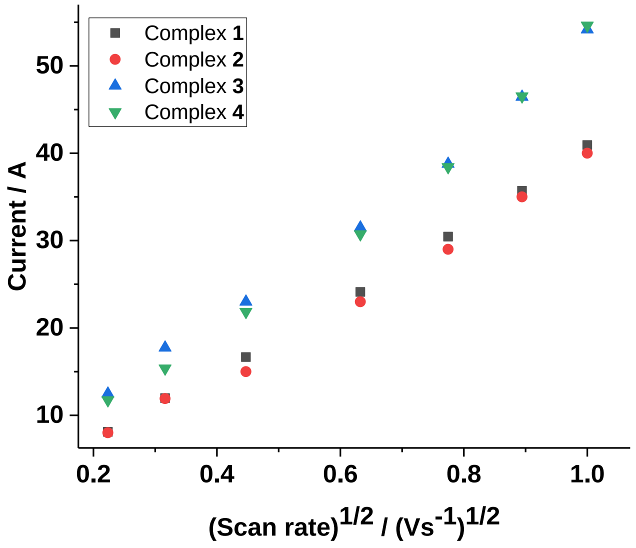

Figure S11. Plots of current vs square root of scan rate (0.05-1 VS^{-1}) (first reduction) for complexes **1-4**.

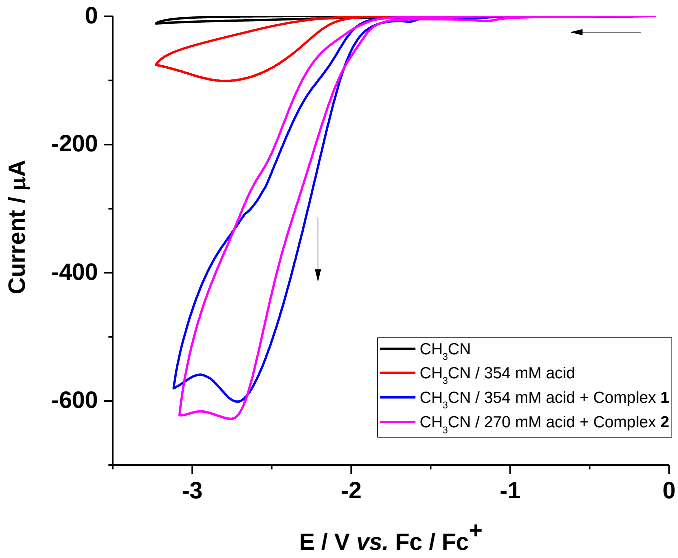

Figure S12. CVs for CH_3CN, CH_3CN/acetic acid and CH_3CN/acetic acid/complex at a scan rate of 0.1 V s^{-1}.

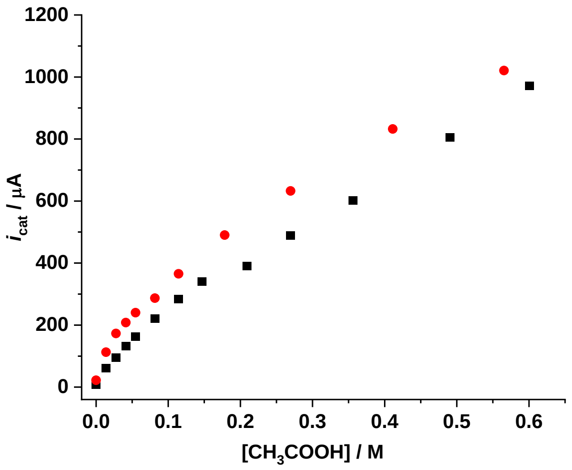

Figure S13. Plots of i_{cat}/µA vs. [CH_3COOH]/M for **3** (1.11 mM) (■) and **4** (1.13 mM) (●) for the second reduction peak at a scan rate of 0.1 Vs^{-1}. The negative sign for the current has been ignored.

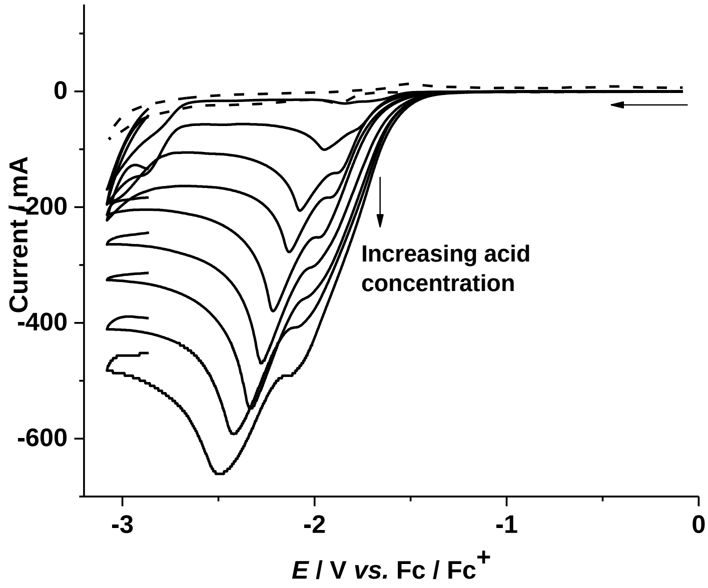

Figure S14. Cyclic voltammograms for complex **1** (1 mM) in acetonitrile without acid (---) and with increasing amounts (1.2-78.2 mM) of TFA (—) at a scan rate of 0.1 Vs^{-1}. Reverse scans have been omitted for clarity.

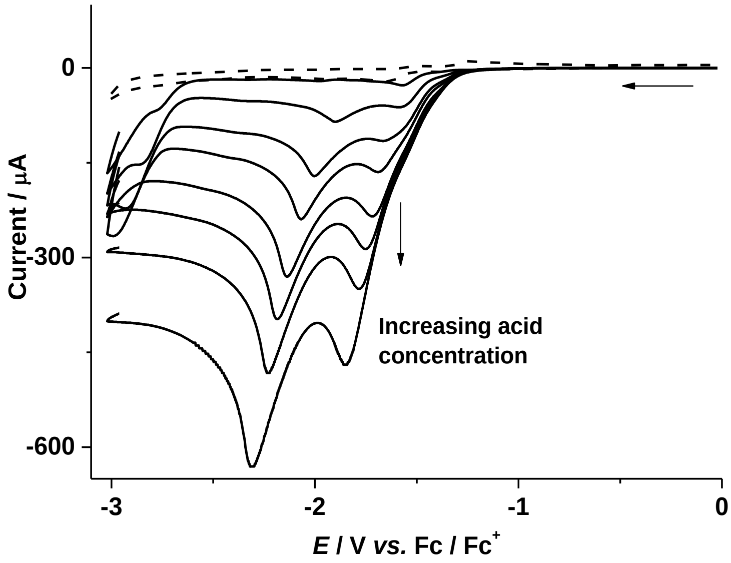

Figure S15. Cyclic voltammograms for complex **3** (1 mM) in acetonitrile without acid (---) and with increasing amounts (1.2-78.2 mM) of TFA (—) at a scan rate of 0.1 Vs^{-1}. Reverse scans have been omitted for clarity.

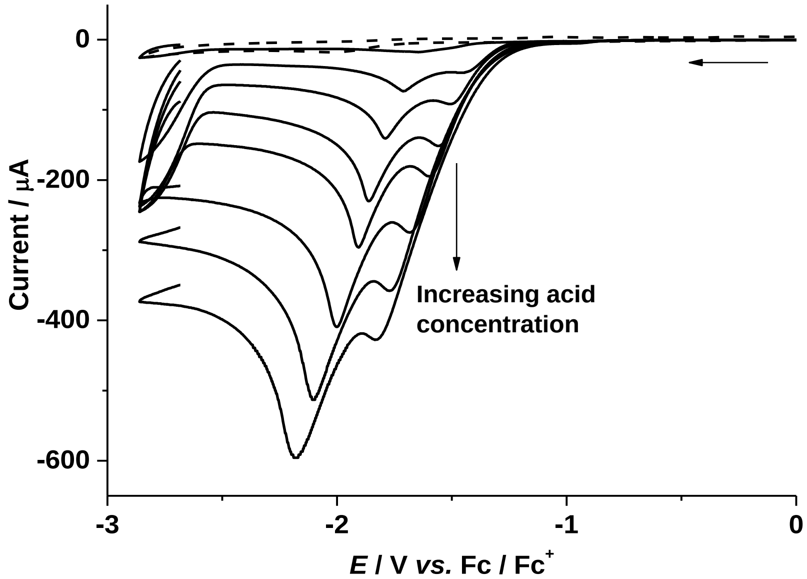

Figure S16. Cyclic voltammograms for complex **4** (0.65 mM) in acetonitrile without acid (----) and with increasing amounts (1.2-64.3 mM) of TFA (—) at a scan rate of 0.1 Vs^{-1}. Reverse scans have been omitted for clarity.

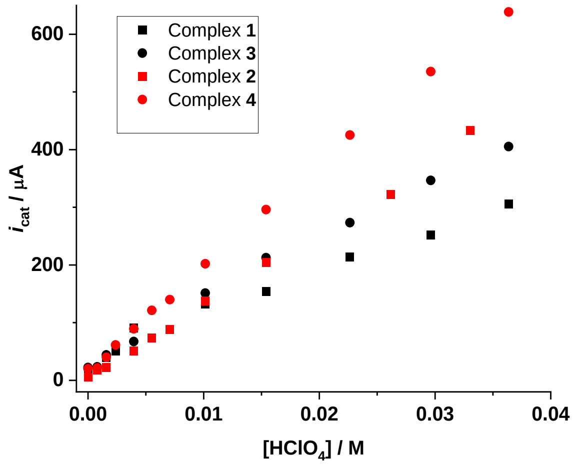

Figure S17. Plots of i_{cat} vs. [$HClO_4$] acid concentration for complexes **1** (■), **2** (■), **3** (•) and **4** (•)at a scan rate of 0.1 Vs^{-1}. The negative sign for the current has been ignored.

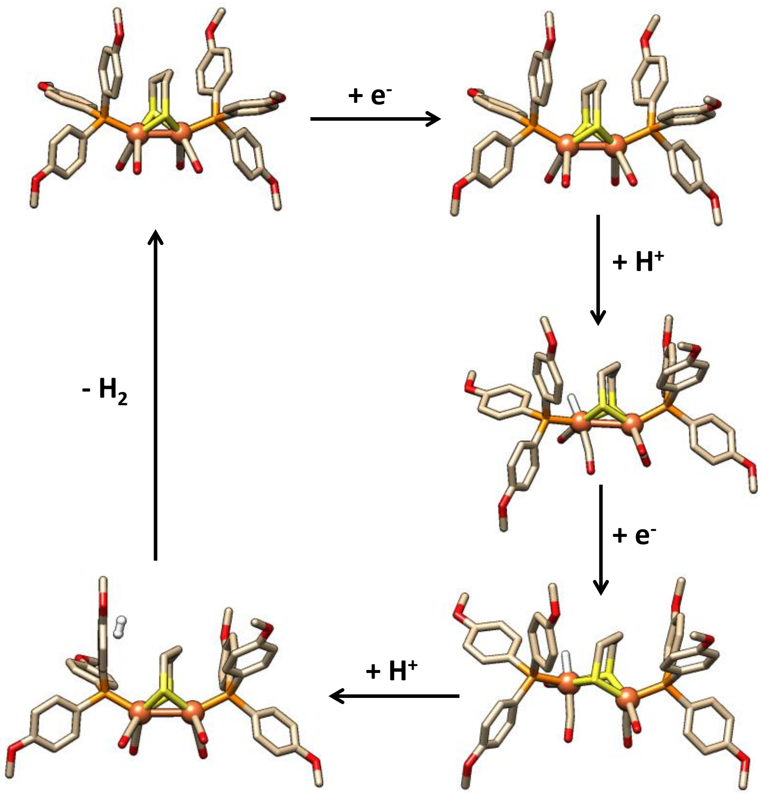

Scheme S1. ECEC Mechanism for Proton Reduction of complex **2** in acidic medium (H atoms of complex have been omitted for clarity).

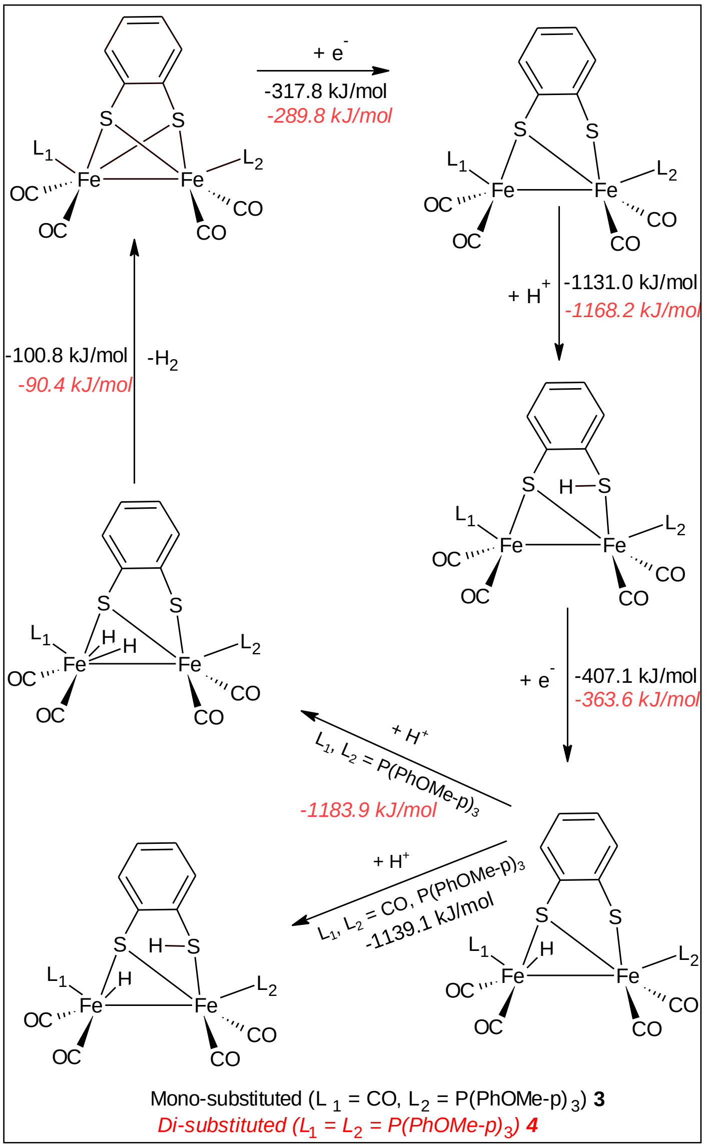

Scheme S2. ECEC Mechanism for Proton Reduction of complex **3** and **4** in acidic medium.

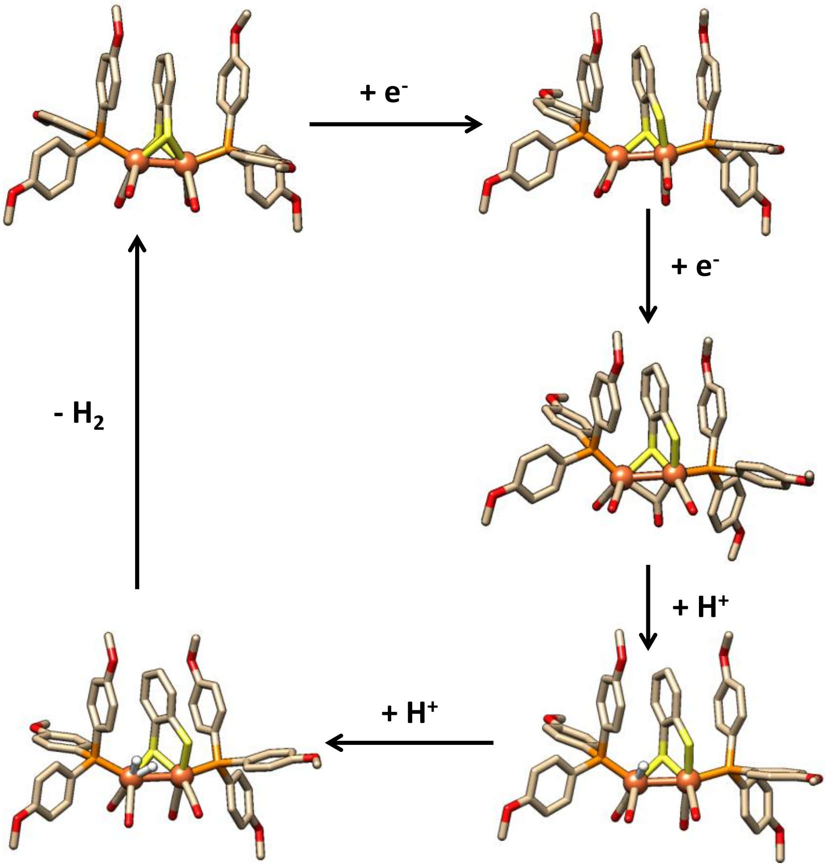

Scheme S3. EECC Mechanism for Proton Reduction of complex **4** in acidic medium (H atoms of complex have been omitted for clarity).

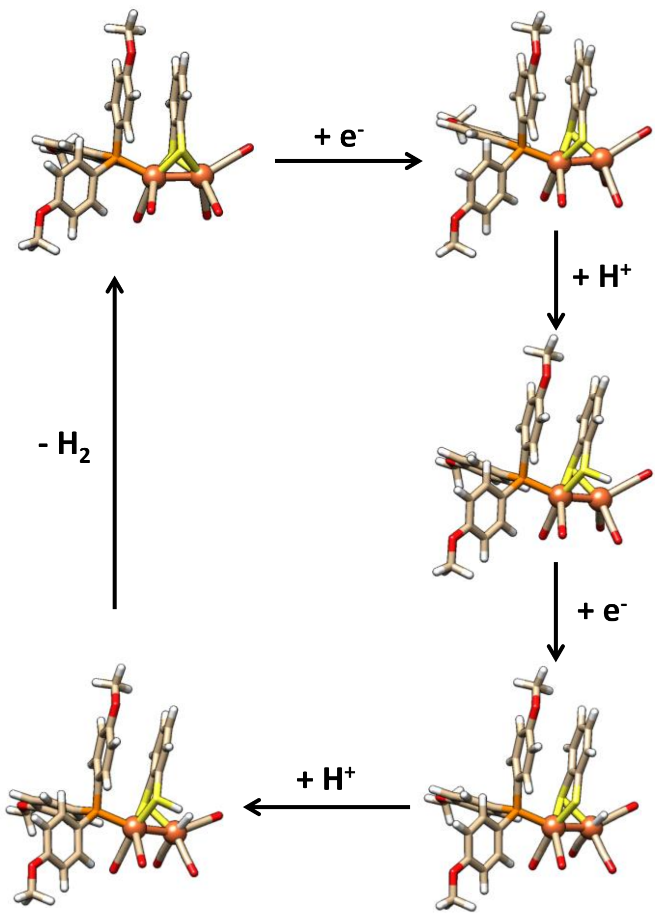

Scheme S4. ECEC Mechanism for Proton Reduction of complex **3** in acidic medium.

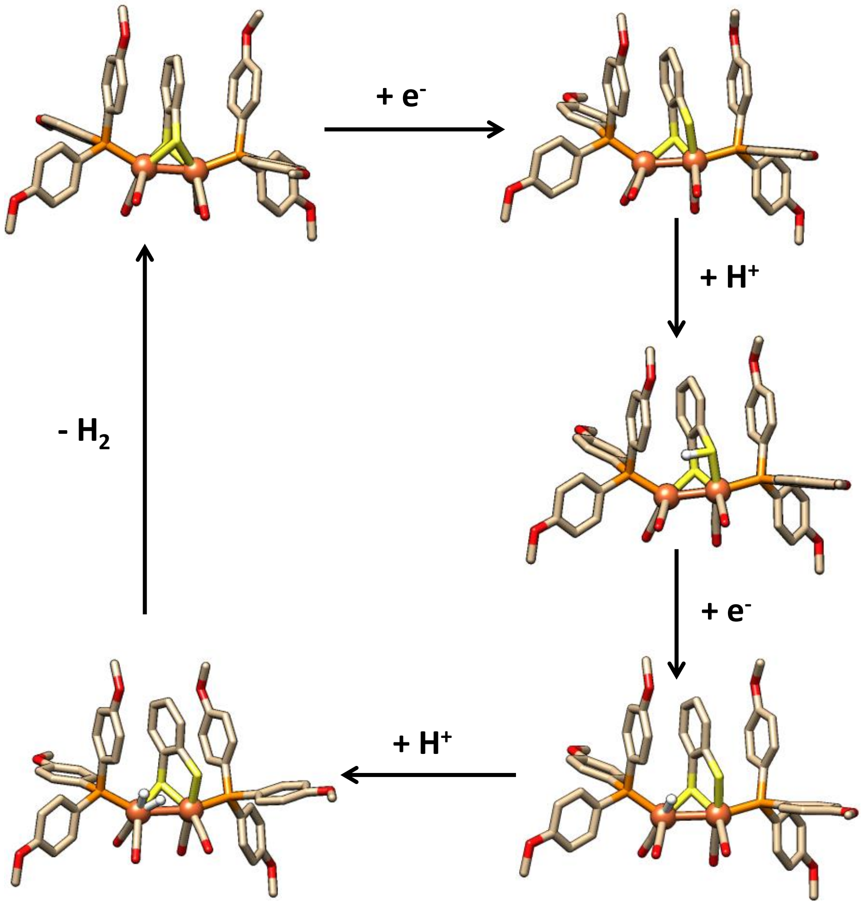

Scheme S5. ECEC Mechanism for Proton Reduction of complex **4** in acidic medium (H atoms of complex have been omitted for clarity).

Table S7. Experimental and B3LYPD3/def2-TZVP calculated reduction potentials.

Complex	E_{pc} / V	E_{pa} / V	E_{cat} / V In presence of TFA	*Calculated* E_{pc} /V
**1**	–1.87<br>–2.37	0.35<br>0.70	–1.79<br>–1.96	–1.19<br>–2.56
**2**	–2.10<br>–2.51	0.27	–1.65<br>–1.99	–1.08<br>–2.73
**3**	–1.65	0.49	–1.57<br>–1.91	–1.72<br>–2.10
**4**	–2.02	0.39<br>0.67	–1.43<br>–1.71	–1.19<br>–2.50
$[Fe_2(CO)_6(\mu\text{-pdt})]$[a]	–1.74<br>–2.35	-	-	–0.84<br>–1.51
$[Fe_2(CO)_6(\mu\text{-bdt})]$	–1.31[b]	-	-	–1.11

[a]in dichloromethane, [b] $E_{1/2}^{\mathrm{red}}$

Table S8. Energy and Gibbs free energy (ΔE and ΔG in kJ/mol) of formation of each intermediate in the HER using complex $[Fe_2(CO)_6(\mu\text{-pdt})]$ **A** in absence and presence of solvent.

**A**	BP86-D3/def2-TZVP				B3LYP-D3/def2-TZVP			
	Vacuum		Solvent		Vacuum		Solvent	
**(ECEC)**	ΔE	ΔG	ΔE	ΔG	ΔE	ΔG	ΔE	ΔG
**$A + e^- \rightarrow A^-$**	-182.5	-193.2	-319.2	-329.9	-163.1	-177.2	-299.2	-313.4
**$A^- + H^+ \rightarrow FeH$**	-1279.7	-1251.3	-1141.7	-1113.3	-1321.1	-1292.3	-1183.4	-1154.6
**$FeH + e^- \rightarrow FeH^-$**	-213.7	-215.9	-356.3	-358.4	-167.8	-168.5	-308.0	-308.7
**$FeH^- + H^+ \rightarrow A.H_2$**	-1417.1	-1412.4	-1275.6	-1270.9	-1429.6	-1428.3	-1290.6	-1289.3
**$A.H_2 \rightarrow A + H_2$**	-4.5	22.1	-3.3	23.3	-4.9	16.2	-3.6	17.4
**Other possible mechanism for the last two steps**								
**$FeH^- + H^+ \rightarrow FeHSH$**	-1259.9	-1233.0	-1142.9	-1116.0	-1274.4	-1245.8	-1163.1	-1134.5
**$FeHSH \rightarrow A + H_2$**	-152.7	-201.5	-129.4	-178.2	-150.3	-198.7	-123.9	-172.2

Table S9. Energy and Gibbs free energy (ΔE and ΔG in kJ/mol) of formation of each intermediate in the HER using complex $[Fe_2(CO)_5(\mu\text{-pdt})(P(PhOMe\text{-}p)_3)]$ **1** in absence and presence of solvent.

**1**	BP86-D3/def2-TZVP				B3LYP-D3/def2-TZVP			
	Vacuum		Solvent		Vacuum		Solvent	
**(ECEC)**	ΔE	ΔG	ΔE	ΔG	ΔE	ΔG	ΔE	ΔG
**1 + e$^-$ → 1$^-$**	-147.6	-150.3	-295.1	-297.8	-132.0	-154.1	-284.3	-306.4
**1$^-$ + H$^+$ → 1FeH**	-1326.4	-1297.6	-1176.0	-1147.3	-1365.7	-1329.8	-1210.5	-1174.6
**1FeH + e$^-$ → 1FeH$^-$**	-152.7	-164.9	-294.0	-306.2	-95.4	-101.6	-239.9	-246.1
**1FeH$^-$ + H$^+$ → 1.H$_2$**	-1458.4	-1444.8	-1320.9	-1307.3	-1477.9	-1469.4	-1336.5	-1328.0
**1.H$_2$ → 1 + H$_2$**	3.5	37.3	3.4	37.2	5.8	27.7	6.4	28.3
				**Other possible mechanism for the last two steps**				
**1FeH$^-$ + H$^+$ → 1FeHSH**	-1283.9	-1248.3	-1159.1	-1123.6	-1299.7	-1266.0	-1176.1	-1142.4
**1FeHSH → 1 + H$_2$**	-178.0	-233.7	-165.2	-220.9	-184.0	-231.1	-166.8	-213.9

Table S10. Energy and Gibbs free energy (ΔE and ΔG in kJ/mol) of formation of each intermediate in the HER using complex $[Fe_2(CO)_4(\mu\text{-pdt})(P(PhOMe\text{-}p)_3)_2]$ **2** in absence and presence of solvent.

**2**	BP86-D3/def2-TZVP				B3LYP-D3/def2-TZVP			
	Vacuum		Solvent		Vacuum		Solvent	
**(ECEC)**	ΔE	ΔG	ΔE	ΔG	ΔE	ΔG	ΔE	ΔG
**2 + e$^-$ → 2$^-$**	-98.6	-105.2	-237.5	-244.2	-99.0	-108.2	-246.0	-255.2
**2$^-$ + H$^+$ → 2FeH**	-1380.2	-1345.6	-1239.9	-1205.4	-1399.8	-1373.6	-1250.6	-1224.3
**2FeH + e$^-$ → 2FeH$^-$**	-119.7	-129.6	-270.7	-280.6	-64.6	-71.3	-219.6	-226.3
**2FeH$^-$ + H$^+$ → 2.H$_2$**	-1495.4	-1476.3	-1346.2	-1327.1	-1513.8	-1497.5	-1361.1	-1344.8
**2.H$_2$ → 2 + H$_2$**	-5.3	38.2	-4.8	38.6	-0.5	31.9	0.3	32.7
				**Other possible mechanism for the last two steps**				
**2FeH$^-$ + H$^+$ → 2FeHSH**	-1325.4	-1292.4	-1189.8	-1156.8	-1341.3	-1306.3	-1204.9	-1169.8
**2FeHSH → 2 + H$_2$**	-164.7	-222.1	-151.5	-208.9	-172.0	-223.1	-156.6	-207.7

Table S11. Energy and Gibbs free energy (ΔE and ΔG in kJ/mol) of formation of each intermediate in the HER using complex $[Fe_2(CO)_6(\mu\text{-bdt})]$ **B**, in vacuum and in solvent

**B**	BP86-D3/def2-TZVP				B3LYP-D3/def2-TZVP			
	Vacuum		Solvent		Vacuum		Solvent	
	ΔE	ΔG	ΔE	ΔG	ΔE	ΔG	ΔE	ΔG
				**EECC Mechanism**				
**B + e$^-$ → B$^-$**	-194.1	-209.4	-329.5	-344.8	-185.9	-204.5	-321.1	-339.6
**B$^-$ + e$^-$ → B^{2-}**	78.3	81.5	-352.0	-348.8	149.6	156.0	-281.3	-274.9
**B^{2-} + H$^+$ → FeH$^-$**	-1335.5	-1310.7	-782.7	-757.8	-1424.4	-1401.7	-870.6	-847.9
**FeH$^-$ + H$^+$ → FeH$_2$**	-1310.5	-1284.7	-1165.1	-1139.2	-1349.7	-1322.9	-1208.9	-1182.1
**FeH$_2$ → B + H$_2$**	-57.2	-102.3	-55.1	-100.3	-67.5	-114.6	-61.5	-108.5
				**CE steps of ECEC Mechanism (first protonation and second reduction, rest steps are same as in EECC)**				
**B$^-$ + H$^+$ → SH**	-1257.3	-1229.2	-1134.7	-1106.6	-1274.8	-1245.6	-1151.9	-1122.7
**SH + e$^-$ → FeH$^-$**	-269.6	-269.3	-405.1	-404.8	-198.7	-194.9	-334.2	-330.4

Table S12. Energy and Gibbs free energy (ΔE and ΔG in kJ/mol) of formation of each intermediate in the HER using complex $[Fe_2(CO)_5(\mu\text{-bdt})(P(PhOMe\text{-}p)_3)]$ **3** in absence and presence of solvent.

**3**	BP86-D3/def2-TZVP				B3LYP-D3/def2-TZVP			
	Vacuum		Solvent		Vacuum		Solvent	
	ΔE	ΔG	ΔE	ΔG	ΔE	ΔG	ΔE	ΔG
**EECC Mechanism**								
**3 + e^- → 3^-**	-157.3	-170.7	-304.4	-317.8	-151.1	-172.8	-295.1	-316.8
**3^- + e^- → 3^{2-}**	90.8	90.0	-301.1	-302.0	169.0	184.5	-233.5	-218.1
**3^{2-} +H^+ → 3FeH$^-$**	-1658.1	-1629.7	-1264.6	-1236.1	-1690.5	-1672.3	-1292.1	-1273.9
**3FeH$^-$ + H^+ → FeHSH**	-1297.9	-1274.0	-1163.0	- 1139.1	-1321.3	-1296.6	-1183.4	-1158.7
**3FeHSH → 3 + H_2**	-66.1	-110.6	-56.4	-100.8	-82.8	-125.3	-73.5	-115.9
**CE steps of ECEC Mechanism (first protonation and second reduction, rest steps are same as in EECC)**								
**3^- + H^+ → 3SH**	-1295.2	-1264.6	-1161.7	-1131.0	-1309.6	-1277.0	-1178.2	-1145.5
**3SH + e^- → 3FeH$^-$**	-272.1	-275.1	-404.0	-407.1	-211.9	-210.8	-347.4	-346.4

Table S13. Energy and Gibbs free energy (ΔE and ΔG in kJ/mol) of formation of each intermediate in the HER using complex $[Fe_2(CO)_4(\mu\text{-bdt})(P(PhOMe\text{-}p)_3)_2]$ **4** in absence and presence of solvent.

**4**	BP86-D3/def2-TZVP				B3LYP-D3/def2-TZVP			
	Vacuum		Solvent		Vacuum		Solvent	
	ΔE	ΔG	ΔE	ΔG	ΔE	ΔG	ΔE	ΔG
**EECC**								
**4 + e^- → 4^-**	-139.8	-150.6	-279.0	-289.8	-147.3	-166.9	-286.6	-306.2
**4^- + e^- → 4^{2-}**	97.1	88.9	-262.7	-270.9	186.7	183.6	-176.8	-179.9
**4^{2-} + H^+ → 4FeH$^-$**	-1650.9	-1619.4	-1292.5	-1260.9	-1502.3	-1476.1	-1013.7	-987.4
**4FeH$^-$ + H^+ → 4FeH$_2$**	-1358.4	-1330.4	-1211.9	-1183.9	-1377.4	-1345.6	-1237.3	-1205.5
**4FeH$_2$ → 4 + H_2**	-36.5	-83.5	-43.4	-90.4	-50.9	-98.4	-50.3	-97.8
**CE steps of ECEC Mechanism (first protonation and second reduction, rest steps are same as in EECC)**								
**4^- + H^+ → 4SH**	-1317.0	-1294.3	-1190.8	-1168.2	-1315.7	-1292.5	-1190.4	-1167.3
**4SH + e^- → 4FeH$^-$**	-236.9	-236.1	-364.4	-363.6	-185.4	-179.1	-313.0	-306.7

References

[1] (a) Javier A. Cabeza, M. Angeles Martınez-Garcıa, Vıcto Rier, *Organometallics*, **1998**, *17*, 1471-1477; (b) M. M. Hasan, M. B. Hursthouse, S. E. Kabir, K. M. Abdul Malik, *Polyhedron* ***2001***, *20*, 97-101.

[2] P.-H. Zhao, X.-H. Li, Y.-F. Liu, Y.-Q. Liu, *J. Coord. Chem.* **2014**, *67*, 766-778.
